# The Immunologic Effects of Mesalamine in Treated HIV-Infected Individuals with Incomplete CD4+ T Cell Recovery: A Randomized Crossover Trial

**DOI:** 10.1371/journal.pone.0116306

**Published:** 2014-12-29

**Authors:** Ma Somsouk, Richard M. Dunham, Michelle Cohen, Rebecca Albright, Mohamed Abdel-Mohsen, Teri Liegler, Jeffrey Lifson, Michael Piatak, Robert Gorelick, Yong Huang, Yuaner Wu, Priscilla Y. Hsue, Jeffrey N. Martin, Steven G. Deeks, Joseph M. McCune, Peter W. Hunt

**Affiliations:** 1 Division of Gastroenterology, Department of Medicine, University of California San Francisco, San Francisco, CA, 94110, United States of America; 2 Division of Experimental Medicine, Department of Medicine, University of California San Francisco, San Francisco, CA, United States of America; 3 Positive Health Program, Department of Medicine, University of California San Francisco, San Francisco, CA, United States of America; 4 AIDS and Cancer Virus Program, Leidos Biomedical Research Inc., Frederick National Laboratory, Frederick, MD, United States of America; 5 Division of Cardiology, Department of Medicine, University of California San Francisco, San Francisco, CA, United States of America; Asociacion Civil Impacta Salud y Educacion, Peru

## Abstract

The anti-inflammatory agent, mesalamine (5-aminosalicylic acid) has been shown to decrease mucosal inflammation in ulcerative colitis. The effect of mesalamine in HIV-infected individuals, who exhibit abnormal mucosal immune activation and microbial translocation (MT), has not been established in a placebo-controlled trial. We randomized 33 HIV-infected subjects with CD4 counts <350 cells/mm^3^ and plasma HIV RNA levels <40 copies/ml on antiretroviral therapy (ART) to add mesalamine vs. placebo to their existing regimen for 12 weeks followed by a 12 week crossover to the other arm. Compared to placebo-treated subjects, mesalamine-treated subjects did not experience any significant change in the percent CD38+HLA-DR+ peripheral blood CD4+ and CD8+ T cells at week 12 (P  = 0.38 and P  = 0.63, respectively), or in the CD4+ T cell count at week 12 (P  = 0.83). The percent CD38+HLA-DR+ CD4+ and CD8+ T cells also did not change significantly in rectal tissue (P  = 0.86, P  = 0.84, respectively). During the period of mesalamine administration, plasma sCD14, IL-6, D-dimer, and kynurenine to tryptophan ratio were not changed significantly at week 12 and were similarly unchanged at week 24. This study suggests that, at least under the conditions studied, the persistent immune activation associated with HIV infection is not impacted by the anti-inflammatory effects of mesalamine.

**Trial Registration:**

ClinicalTrials.gov NCT01090102

## Introduction

Despite effective antiretroviral therapy (ART), HIV-infected individuals – particularly those with incomplete CD4+ T cell recovery on ART - continue to have a shorter life expectancy than the general population and remain at higher risk for morbidities that are normally associated with aging. [1], [2], [3], [4] Increased immune activation has been linked to microbial translocation (MT) during both untreated and treated HIV infection. [5], [6], [7], [8] Observations in pathogenic simian immunodeficiency virus infection and HIV infection underscore the relationship of such MT with disruption of mucosal immune and intestinal epithelial homeostasis. [9], [10], [11] Since persistent inflammation appears to be a major mediator of this increased risk of morbidity and mortality, and since epithelial barrier dysfunction persists during treated HIV infection, interventions targeting the mucosal lining and serving to decrease immune activation warrant investigation. [12], [13], [14], [15], [16], [17].

In the setting of inflammatory bowel disease, several lines of evidence suggest that anti-inflammatory agents that improve gut mucosal integrity can reduce mucosal and systemic inflammation. [18], [19], [20], [21], [22] Although systemic non-steroidal anti-inflammatory drugs (NSAID) have been shown to decrease inflammation during HIV infection, [23], [24], [25] the toxicities associated with chronic systemic NSAIDs (i.e., renal toxicity, liver toxicity, gastrointestinal bleeding, and cardiovascular events) limit their widespread use in HIV-infected individuals. Also, since NSAIDs are distributed systemically, their mucosal effect versus systemic effect would not be distinguishable. In this study, we reasoned that the persistent inflammation found during treated HIV infection and associated with persistent intestinal epithelial barrier dysfunction might be more effectively suppressed, with lower risk to the patient, by a locally bioactive anti-inflammatory agent.

Given its safety profile and efficacy in the treatment of mild to moderately active ulcerative colitis, we sought to examine the effect of mesalamine (5-aminosalicylic acid) on immune activation during chronic HIV infection. We hypothesized that mesalamine would decrease GALT inflammation in HIV-infected individuals, blocking the vicious cycle of local HIV replication, Th17 depletion, epithelial damage and MT, thereby resulting in a decreased level of systemic T cell activation. To test this possibility, we performed a randomized placebo-controlled trial of mesalamine among HIV-infected subjects maintaining ART-mediated viral suppression. We focused on individuals with incomplete CD4+ T cell recovery (CD4 count<350 cells/mm^3^) as they tend to have the highest levels of persistent immune activation and are at highest risk for morbidity and mortality. Our *a priori* hypothesis was that 12 weeks of mesalamine would reduce systemic CD8+ T cell activation in this setting. We also performed serial rectal biopsies on a subset of participants to determine the effects of mesalamine on gut-associated lymphoid tissue (GALT). [26].

## Methods

### Trial design and study subjects

Enrolled subjects on continuous suppressive ART were randomized to receive either mesalamine or matching placebo for 12 weeks, followed by a 12 week crossover period on the alternative arm. The primary outcome was the change in the percent activated (CD38+ HLA-DR+) CD8+ T cells at week 12. Consenting subjects also participated in a serial rectal biopsy sub-study to evaluate the effects of mesalamine in the GALT.

Subjects were recruited from the University of California, San Francisco [UCSF] between February, 2011 and June, 2012 with the last date of follow-up occurring in December, 2012. Chronically HIV-infected adults maintaining plasma HIV RNA levels below the limit of detection of the locally available clinical threshold for ≥1 year on stable ART and with persistent CD4+ T cell counts <350 cells/mm^3^ were eligible. Detectable episodes of viremia <500 copies/ml were allowed in the prior year if they were flanked by confirmed undetectable values. Patients were ineligible if they experienced an increase in CD4+ T cell count >100 cells/mm^3^ in the last year; reported <90% adherence to ART; had any serious acute illness in the preceding 3 months; were pregnant or breastfeeding; or had any of the following laboratory abnormalities: absolute neutrophil count <1,000 cells/mm^3^, platelet count <50,000 cells/mm^3^, hemoglobin <8 mg/dL, creatinine clearance <40 mL/minute, or serum transaminases >2.5x the upper limit of normal. For the endothelial function studies, as nitroglycerin is administered, any Viagra-like drug within 72 hours of the study was prohibited. Changes in doses of lipid lowering medication or anti-hypertensive medication were also not permitted less than 90 days prior to study entry or during the study.

The study was approved by the institutional review board at UCSF and all subjects provided written informed consent (IRB #: 10–010123; Clinical Trials #: NCT01090102). The protocol for this trial and supporting checklist are available as S1 Protocol and S1 CONSORT Checklist.

### Study procedures

Enrolled subjects were randomized study by the study pharmacist (1∶1 ratio, block sizes of 2) to receive mesalamine (Apriso, Salix Pharmaceuticals, NC) (a dose that is normally used to treat ulcerative colitis) [27] or matching placebo (formulated to have the identical appearance and weight), four 375 mg tablets once daily for 12 weeks, followed by a 12-week crossover period during which subjects who initially started on placebo would receive mesalamine and vice versa. Apriso is a locally-acting aminosalicylate indicated for the maintenance of remission of ulcerative colitis in adults. [28], [29] It is a pH-dependent delayed- and extended-release product composed of granules composed of mesalamine in a polymer matrix, which targets the release of the active drug to the terminal ileum and colon [27].

Subjects, coordinators, clinicians, and laboratory personnel were blinded to treatment assignment. Study visits occurred at weeks 2, 4, 8, 12, 14, 16, 20, and 24 from baseline. CD4+ and CD8+ T cell counts, liver function tests, complete blood count with differential, and renal function tests were measured at each study visit. Plasma HIV RNA levels (by clinical assay) were measured at baseline, week 12, and week 24 during the study. Peripheral blood mononuclear cells (PBMC), plasma, and serum were cryopreserved at baseline and weeks 4, 12, 16, and 24 for immunologic, and soluble biomarkers of inflammation, and for low-level viremia testing by single-copy assay.

### Laboratory measurements

T cell immunophenotyping was performed on fresh PBMCs with the primary outcome being the percent CD38+HLA-DR+ CD8+ and CD4+ T cells. Surface staining was performed, followed by fixation and permeabilization, intracellular staining and fixation in 0.5% paraformaldehyde, as per the manufacturers’ protocol (BD Cytofix/Cytoperm, BD Biosciences, San Jose, CA and Foxp3/Transcription Factor Staining Set, eBioscience, San Diego, CA). IL-17 production was evaluated on a subset (1 million) of PBMCs stimulated with PMA/Ionomycin for 18 hours in complete media containing Golgi Stop and Golgi Plug (BD Biosciences, San Jose, CA) and compared to 1 million unstimulated PBMCs. Stained cells were acquired on a BD LSRII flow cytometer with BD FACSDiva software (BD Biosciences, San Jose, CA), and analyzed using FlowJo v9.4 (TreeStar Inc., Ashland, OR). The flow gates were delineated manually by personnel blinded to the treatment status of the study subjects.

Plasma HIV RNA levels at single-copy per test were determined in a triplex, hybrid real time/digital PCR approach based on the gag and internal control assays previously described with details to be described in a manuscript in preparation. [30] Briefly, a triplex assay comprised of the gag and RCAS internal control primer sets referenced above but including an additional gag-based assay targeted to the region encoding the start of gag and consisting of HMMCgagF06, 5′-GCGGCG(dK)C(dP)GGTGAGTACGC, HMMCgagR06, GACGCTCTCG CACCCATCTCT, and HMMCgagP05, FAM-TTGACTAGCG GAGGCTAG-MGB, where the 5′end of the forward primer corresponds to nucleotide 733 in GenBank accession no. M38432 and dP and dK bases are included to facilitate broader sequence coverage of potential virus variants was utilized. [31] Reverse transcription-PCR assays were setup in 12 replicates. Where all reactions showed positive amplifications, viral loads were determined by interpolation on a standard curve; where less than all reactions showed positive amplification, viral loads were determined by application of Poisson distribution analysis of the determined positive frequency. Plasma volumes of 9 mL were typically processed for RNA extraction and 80% of the total sample tested resulting in a threshold sensitivity for 1 of 12 positive determinations of 0.15 copies per mL.

### Plasma biomarkers of inflammation

Plasma was stored at −80°C prior to batch testing. High sensitivity ELISA kits were used to quantitate levels of IL-6 (R&D Systems), D-dimer (Diagnostica-Stago), and soluble CD14 (sCD14, R&D Systems), according to the manufacturers’ instructions. Plasma concentrations of kynurenine and tryptophan were determined by liquid chromatography and mass spectroscopy [32].

### Endothelial function and hyperemic velocity studies

As previously described, [33], [34] we used a 10 MHz linear array vascular ultrasound probe coupled to a GE Vivid7 Imaging Console. To assess endothelium-dependent dilation, the forearm cuff was inflated to 250 mmHg for 5 minutes. After deflation, spectral Doppler images are obtained for the first 15 seconds to assess reactive hyperemia (RH) and B-mode ultrasound is performed between 30–120 seconds every 15 seconds to assess FMD. [35], [36] ^,^ [37] The percent FMD was calculated as the ratio between the maximum post cuff release brachial artery diameter and the baseline diameter. Microvascular function was assessed as maximal RH from the Doppler mean velocity-time integral (VTI) of the first 3 complete beats after cuff release. Independently of FMD, RH strongly correlates with CVD risk. [35], [36] To assess endothelium-independent dilation, after 10 minutes of rest, brachial artery dilation was determined under basal conditions and following the administration of sublingual nitroglycerin (0.4 mg). Analysis of digitized images is performed using dedicated software (Medical Imaging Applications, LLC, Coralville, IA). The individual performing image analysis was blinded to the subject’s treatment status. We have previously repeated brachial artery reactivity studies on 25 HIV-infected individuals to show that the intra-observer reliability for measurement of FMD was 0.976, which reflects an interclass correlation coefficient and the coefficient of variation for FMD was 2.7%, demonstrating a high level of reproducibility.

### Rectal biopsy sub-study

Consenting subjects underwent flexible sigmoidoscopy with rectal biopsies 2 weeks prior to baseline, and at weeks 10 (2 weeks before primary endpoint) and 22 (2 weeks before end of study). Biopsies of rectal mucosa (approximately 3 mm×2 mm×2 mm) were obtained at 10–20 cm above the anus using jumbo forceps. Eighteen biopsies were placed immediately in 15 ml of RPMI 1640 with 10% fetal calf serum, with piperacillin–tazobactam (500 µg/ml), and amphotericin B (1.25 µg/ml), and transported within 1 hour to the Division of Experimental Medicine at UCSF, where they were processed the same day. [38] Rectal mononuclear cells (RMC) were isolated from biopsy specimens using a protocol optimized for lymphocyte viability and yield. [39] Biopsy pieces were digested with three rounds of 0.5 mg/mL collagenase type II (Sigma-Aldrich), and the tissue was then disrupted with a syringe bearing a 16-gauge blunt-end needle and passaged through a 70 µm cell strainer. RMC were stained with antibodies as described above.

Between one and three frozen rectal lymphoid tissue pieces were homogenized using a TissueLyser II (Qiagen, Ventura CA). Total cellular RNA and DNA was immediately isolated using the AllPrep DNA/RNA kit (Qiagen) as specified by the manufacturer. Cellular DNA and RNA was quantified using a Nanodrop *(*ND-1000*)* spectrophotometer and normalized to cell equivalents by qPCR using human genomic TERT for DNA and GAPDH expression for RNA (Life Technologies, Grand Island NY). Total cellular HIV-1-specific DNA and RNA was quantified with a single copy detection qPCR TaqMan assay [40] using triplicate measurements on a StepOne Plus Real-time PCR System (Applied Biosystems Inc, Foster City CA).

### Statistical analysis

The primary analysis compared the week 12 change from baseline in the log-transformed percent activated CD8+ T cells between mesalamine and placebo-treated subjects with a t-test; secondary analyses for all other measured biomarkers were carried out similarly. Differences between treatment arms in T cell activation changes (and all other biomarker changes) across all time points were also assessed with linear mixed models with compound symmetry covariance structure, log-transforming the outcome if necessary to satisfy model assumptions (xtmixed in Stata 11, College Station, TX). Changes in slopes before and after week 12 were assessed using linear splines. An additional analysis was performed aggregating measurements by treatment and time on mesalamine. Lastly, since the bioactivity of mesalamine may overlap with that of aspirin, an analysis was performed within subjects who reported no aspirin or NSAID use. [41] For all analyses, observations were censored for early discontinuation of study medication. Data derived from this trial are available for public use (S1 Dataset).

### Sample size determination

Based on prior trials of interventions to decrease immune activation in HIV-infected individuals with suboptimal CD4 recovery, we expected a median of 15% activated CD8+ T cells and a standard deviation of the week 12 change in percent activated CD8+ T cells of 3.3%. Assuming this standard deviation and a two-sided Type I error rate of 5%, we would have an 80% chance to detect a difference as small as 3.5% between groups if we enrolled 14 participants into each arm (or a relative difference between groups of 23%). Anticipating up to 10% early discontinuations, the target enrolment was 15 participants in each arm. The trial was over-enrolled by 3 subjects to account for premature treatment discontinuations.

## Results

### Subject characteristics

**Figure 1:**
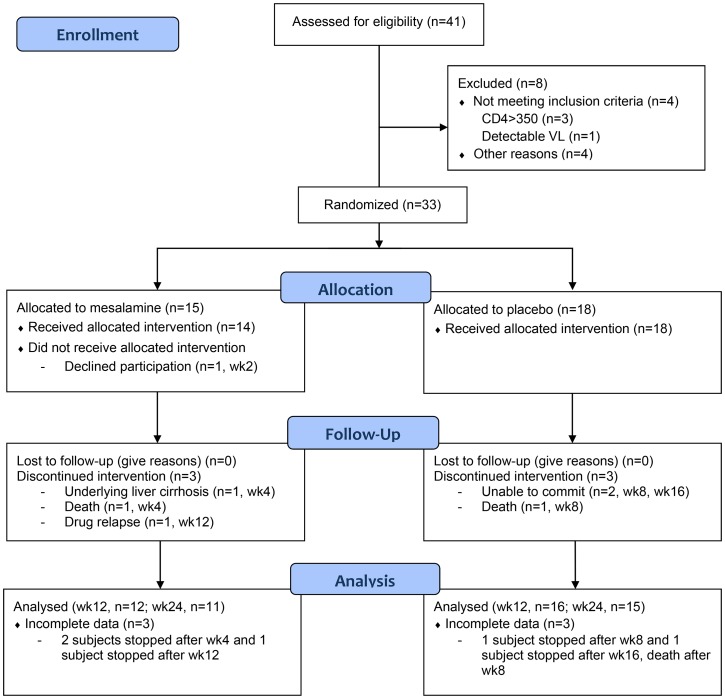
Enrollment, allocation, and follow-up for trial subjects. The outcomes of the 41 screened subjects are described in the flow diagram.

Of 41 screened subjects, 4 did not meet inclusion criteria and 4 did not participate for other reasons (Fig. 1). Baseline characteristics of mesalamine (n  = 15) and placebo-treated (n  = 18) subjects were comparable between arms (Table 1). All were men between the ages of 28 and 68 who had been receiving an ART regimen for a median of 24 months. The median CD4+ T cell count was 247 cells/mm^3^ and all had a plasma HIV RNA level <40 copies/ml at the baseline visit. Aspirin use was reported in 4 (27%) subjects starting on mesalamine and 8 (44%) starting on placebo. A variety of ART regimens were used in the cohort.

**Table 1:** Baseline Characteristics.

Characteristic	Placebo Median (IQR) N = 18	Mesalamine Median (IQR) N = 15
Age, years	60 (49 to 62)	53 (49 to 62)
Male gender, No. (%)	18 (100)	15 (100)
Ethnicity, No. (%)		
White/European-American	14 (78)	13 (87)
Black/African-American	1 (6)	0 (0)
Hispanic/Latino	3 (17)	1 (7)
Asian	0 (0)	1 (7)
CD4+ T cell count, cells/mm^3^	242 (205 to 293)	249 (135 to 269)
Self-reported nadir CD4 count, cells/mm^3^	21 (7 to 50)	39 (14 to 90)
Plasma HIV RNA level, copies/ml	<40	<40
Duration of current ART regimen, months	23 (18 to 34)	31 (17 to 41)
Hepatitis B or C positive, No. (%)	3 (17)	3 (20)
Serum AST level, mg/dl	34 (29 to 39)	35 (25 to 45)
Serum ALT level, mg/dl	37 (26 to 38)	34 (24 to 38)
ART Regimen, No. (%)		
NRTIs+NNRTI	5 (28)	1 (7)
NRTIs+PI	5 (28)	3 (20)
NRTIs+INSTI	2 (11)	0 (0)
3 or more classes	6 (33)	11 (73)
Aspirin use, No. (%)	8 (44)	4 (27)

ART, antiretroviral therapy; NRTI, nucleoside reverse transcriptase inhibitors; NNRTI, non-nucleoside reverse transcriptase inhibitors; PI, protease inhibitor; INSTI, integrase strand transfer inhibitor; AST, aspartate transaminase; ALT, alanine transaminase.

### Study adherence and follow-up

Overall adherence levels by pill count for the placebo and mesalamine at week 12 and after crossover were high. At week 12, adherence to mesalamine was 96.4% (IQR, 93.8%–99.4%) and to placebo was 99.2% (IQR, 97.6%–99.4%). At week 24, adherence to mesalamine was 97.6% (IQR, 92.9%–99.3%) and to placebo was 95.9% (IQR, 90.2%–98.1%). Overall, mesalamine was well tolerated. Twenty-eight of 33 (85%) subjects completed 12 weeks of the study and had specimens to contribute to the primary analysis, and 26 of 33 (79%) subjects completed 24 weeks of the study.

### Impact of mesalamine on immune activation and inflammatory markers

**Figure 2:**
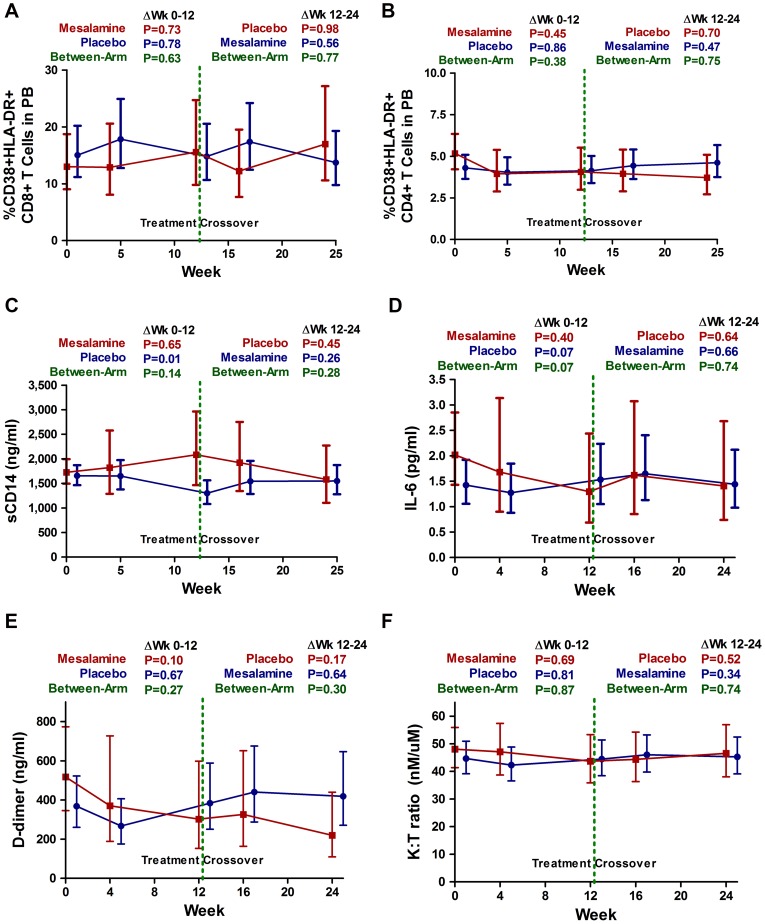
Changes in peripheral blood T cell activation and soluble markers of inflammation during mesalamine. Estimated mean changes (with 95% CI) in the frequency of activated (CD38+ HLA-DR+) CD8+ (**A**) and CD4+ (**B**) T cells, sCD14 (**C**), IL-6 (**D**), D-dimer (**E**), and kynurenine to tryptophan (K:T) ratio (**F**) in peripheral blood are plotted for placebo-treated (blue) and mesalamine-treated (red) subjects. P values are provided for the changes within each indicated interval both within arms and between arms.

Subjects randomized to receive mesalamine in the first 12 weeks had a baseline median percent activated CD8+ T cell levels similar to those randomized to placebo (12.2% vs. 13.4%) (Table 2). Subjects taking mesalamine over the first 12 weeks had no evidence for a change in CD8+ T cell activation, and no evidence for a difference from the placebo arm at any time point (Fig. 2A). There was no evidence for a change in CD8+ T cell activation over the first 12 weeks within either treatment arm (mean change −0.85%, P  = 0.78 in placebo; 2.94%, P  = 0.73 in mesalamine), or a difference between treatment arms (P  = 0.63) (Table 2). During the crossover period, there continued to be no evidence for a change in CD8+ T cell activation over the second 12 weeks within either treatment arm (P  = 0.98 in subjects switching to placebo, P  = 0.56 in subjects switching to mesalamine) (Table 3), or when the mesalamine treatment periods were combined (P  = 0.99) (Table 4). There was also no evidence for an effect of mesalamine when excluding subjects receiving daily aspirin therapy after combining mesalamine treatment time points (data not shown).

**Table 2:** Changes in immunologic, virologic, and cardiovascular parameters during the first 12 weeks of study.

	Placebo (n = 18)			Mesalamine (n = 15)			Between-Arm
	BaselineMedian (%)	Mean log10 Δfrom Wk 0–12(95% CI)	PValue	BaselineMedian (%)	Mean log10 Δfrom Wk 0–12(95% CI)	PValue	PValue
**PERIPHERAL BLOOD**							
*Immunology*							
CD38+HLA-DR+CD8+ T Cells	13.4	−0.01 (−0.10 to 0.07)	0.78	12.2	0.03 (−0.14 to 0.20)	0.73	0.63
CD38+HLA-DR+CD4+ T Cells	3.74	−0.006 (−0.07 to 0.06)	0.86	4.97	−0.05 (−0.17 to 0.07)	0.45	0.38
FoxP3+CD25+CD4+ T Cells	10.6	0.01 (−0.05 to 0.08)	0.72	10.3	−0.0003 (−0.10 to 0.10)	0.99	0.50
IL-17+CD4+ T Cells	1.13	−0.19 (−0.51 to 0.13)	0.24	1.55	−0.25 (−0.65 to 0.15)	0.22	0.89
sCD14 (ng/ml)	1,730	−0.13 (−0.22 to −0.03)	0.01	1,787	−0.02 (−0.09 to 0.06)	0.65	0.14
IL-6 (pg/ml)	1.37	0.15 (−0.01 to 0.32)	0.07	2.05	−0.07 (−0.24 to 0.09)	0.40	0.07
D-dimer (ng/ml)	336	−0.04 (−0.22 to 0.14)	0.67	673	−0.20 (−0.43 to 0.04)	0.096	0.27
K:T ratio (nM/uM)	43	0.005 (−0.03 to 0.04)	0.81	41	0.01 (−0.04 to 0.06)	0.69	0.87
*Virology*							
HIV RNA single copy assay (copy/ml)	0.6	−0.17 (−0.40 to 0.07)	0.16	2.1	−0.01 (−0.33 to 0.13)	0.39	0.68
*Cardiovascular*							
FMD (%)	2.62	−0.004 (−0.06 to 0.05)	0.87	5.19	−0.04 (−0.02 to 0.10)	0.22	0.27
Brachial artery diameter (mm)	4.82	−0.002 (−0.01 to 0.004)	0.51	4.33	−0.002 (−0.009 to 0.005)	0.56	0.99
Hyperemic velocity (cm)	71.3	0.003 (−0.09 to 0.09)	0.95	81.8	−0.02 (−0.10 to 0.06)	0.60	0.73
RECTAL MUCOSAa							
*Immunology*							
CD8+ T Cells (38+/DR+)	19.2	0.01 (−0.08 to 0.10)	0.82	17.6	−0.01 (−0.11 to 0.09)	0.83	0.80
CD4+ T Cells (38+/DR+)	8.7	0.05 (−0.11 to 0.21)	0.57	12.6	0.06 (−0.14 to 0.25)	0.32	0.86
*Virology*							
HIV RNAb (copy per 106 cells)	21.0	0.37 (−0.39 to 1.13)	0.34	15.4	−0.19 (−1.15 to 0.77)	0.70	0.33
HIV DNAb (copy per 106 cells)	52.3	−0.01 (−0.58 to 0.55)	0.97	29.9	0.31 (−0.43 to 1.06)	0.41	0.47

FMD, flow-mediated vasodilation of the brachial artery; K:T, kynurenine:tryptophan; VTI, velocity time integral.

a There were 15 and 11 consented to mucosal biopsies in the placebo arm and mesalamine arm, respectively, but one in each arm did not contribute a second time point.

b Total HIV RNA and DNA level from whole GALT biopsies were based on cell equivalents normalized by GAPDH and TERT copy number, respectively.

**Table 3:** Changes in immunologic, virologic, and cardiovascular parameters during 12 weeks after treatment crossover.

	Placebo → Mesalamine (n = 16)			Mesalamine → Placebo (n = 11)			Between-Arm
	Wk 12Median (%)	Mean log_10_ Δ fromWk 12–24 (95% CI)	P Value	Wk 12Median (%)	Mean log_10_ Δ fromWk 12–24 (95% CI)	P Value	P Value
**PERIPHERAL BLOOD**							
*Immunology*							
CD38+HLA-DR+CD8+ T Cells	13.9	−0.03 (−0.15 to 0.08)	0.56	13.7	0.003 (−0.19 to 0.19)	0.98	0.77
CD38+HLA-DR+CD4+ T Cells	4.3	0.02 (−0.04 to 0.09)	0.47	4.7	0.01 (−0.06 to 0.09)	0.70	0.75
FoxP3+CD25+CD4+ T Cells	9.27	0.01 (−0.05 to 0.08)	0.72	8.66	0.013 (−0.08 to 0.11)	0.80	0.50
IL-17+CD4+ T Cells	0.79	−0.19 (−0.51 to 0.13)	0.24	0.56	−0.15 (−0.60 to 0.29)	0.49	0.89
sCD14 (ng/ml)	1,505	0.08 (−0.06 to 0.21)	0.26	1,855	−0.03 (−0.10 to 0.04)	0.45	0.28
IL-6 (pg/ml)	1.27	−0.04 (−0.20 to 0.12)	0.66	1.86	0.025 (−0.08 to 0.13)	0.64	0.74
D-dimer (ng/ml)	409	0.04 (−0.12 to 0.19)	0.64	459	−0.08 (−0.24 to 0.09)	0.17	0.30
K:T ratio (nM/uM)	44	0.008 (−0.04 to 0.06)	0.34	43	0.01 (−0.03 to 0.06)	0.52	0.74
*Virology*							
HIV RNA single copy assay (copy/ml)	0.7	−0.11 (−0.34 to −0.12)	0.36	1.7	0.04 (−0.21 to 0.28)	0.78	0.43
*Cardiovascular*							
FMD (%)	4.41	−0.02 (−0.11 to 0.07)	0.68	6.33	−0.79 (−0.17 to 0.008)	0.08	0.45
Brachial artery diameter (mm)	4.77	−0.001 (−0.007 to 0.004)	0.64	4.38	−0.005 (−0.01 to 0.002)	0.18	0.41
Hyperemic velocity (cm)	71.3	0.012 (−0.09 to 0.11)	0.81	77.2	0.17 (0.11 to 0.23)	<0.01	0.09
**RECTAL MUCOSA**							
*Immunology*							
CD8+ T Cells (38+/DR+)	24.7	0.02 (−0.09 to 0.14)	0.66	21.8	0.03 (-.08 to 0.14)	0.83	0.91
CD4+ T Cells (38+/DR+)	8.7	0.05 (−0.14 to 0.23)	0.57	12.1	−0.15 (−0.39 to 0.09)	0.21	0.86
*Virology*							
HIV RNA (copy per 10^6^ cells)	22.2	0.18 (−0.80 to 1.16)	0.72	40.6	0.10 (−1.20 to 1.39)	0.88	0.91
HIV DNA (copy per 10^6^ cells)	49.2	−0.14 (−0.51 to 0.24)	0.47	55.7	−0.10 (−0.33 to 0.14)	0.41	0.92

**Table 4:** Changes in T cell activation and soluble markers aggregating the 12 weeks on mesalamine.

	Mesalamine (n = 27)		
Biomarker	Baseline Median (%)	Mean Δ in log_10_% (95% CI)	P Value
**CD38+HLA-DR+38+ CD8+ T Cells**	13.9	0.0003 (−0.09 to 0.09)	0.99
**CD38+HLA-DR+38+ CD4+ T Cells**	4.4	0.01 (−0.06 to 0.09)	0.73
**IL-17+CD4+ T cells**	0.93	−0.10 (−0.32 to 0.12)	0.37
**FoxP3+CD25+ CD4+ T cells**	9.68	0.03 (−0.05 to 0.10)	0.48
**CD4+ T cell** (cells/mm^3^)	246	0.001 (−0.03 to 0.04)	0.95
**sCD14** (ng/ml)	1,604	0.008 (−0.08 to 0.09)	0.86
**IL-6** (pg/ml)	1.46	−0.05 (−0.16 to 0.06)	0.37
**D-dimer** (ng/ml)	480	−0.02 (−0.14 to 0.09)	0.69
**K:T ratio**	41	0.008 (−0.02 to 0.04)	0.59
**FMD (%)**	4.78	0.007 (−0.05 to 0.07)	0.83
**Brachial artery diameter** (mm)	4.55	−0.002 (−0.006 to 0.002)	0.38
**Hyperemic velocity** (cm)	73.1	−0.002 (−0.08 to 0.07)	0.96
**Viral load** (copy/ml)	0.86	−0.11 (−0.28 to 0.07)	0.23

Results for percent CD8+ and CD4+ T cell activation (Fig. 2), Treg, and Th17 also showed no effect of mesalamine at any time point (Table 2 & 3). Similarly, there was no evidence for an effect of mesalamine on sCD14, IL-6, D-dimer, or the ratio of kynurenine/tryptophan (KT) at any time point (Table 2 & 3). While sCD14 levels appeared to decline significantly in the placebo arm in the first 12 weeks of therapy (mean −0.13 log_10_ ug/ml, P  = 0.01), these changes were not observed in the mesalamine arm (P  = 0.65), and there was no evidence for further changes in sCD14 after switching to mesalamine between weeks 12 and 24. In the analysis restricted to individuals who were not taking aspirin or NSAIDs, we did not observe an effect of mesalamine on biomarkers of T cell activation or innate immune activation after combining mesalamine treatment time points (data not shown).

### Effect of mesalamine in the rectal mucosa

A total of 24 subjects (n  = 14 placebo, n  = 10 mesalamine) completed rectal biopsies prior the baseline visit and at week 10 of therapy. Of these, 22 (13 switching to mesalamine, 9 switching to placebo) completed a third rectal biopsy at week 22. The median baseline percent activated CD8+ T cells in rectal mucosa was similar between mesalamine- and placebo-treated subjects prior to the baseline visit (17.6% vs. 19.2%) (Table 2 & 3). There was no evidence for a change in rectal CD8+ T cell activation in either arm between baseline and week 10 (P  = 0.82 in placebo, P  = 0.83 in mesalamine) or between weeks 10 and 22, with no evidence for a difference between arms at any timepoint.

### Changes in virology measurements in gut and peripheral blood

HIV RNA levels were assessed using a single-copy assay at baseline, week 12, and week 24. At baseline, 15 of 18 (83%) placebo-treated subjects and 15 of 15 (100%) of mesalamine-treated subjects had detectable viremia (median 0.6 copies/ml and 2.1 copies/ml, respectively) (Table 2). During both the first 12 weeks and second 12 weeks of the study, there was no evidence for a change in low-level viremia within or between arms. Similarly, the level of HIV RNA, DNA, or RNA/DNA ratio in whole gut tissue was not changed during the study (Table 2 & 3).

### Changes in flow-mediated dilation (FMD) of the brachial artery

At baseline, subjects randomized to start with mesalamine had similar median FMD levels to those starting placebo (2.62% vs. 5.19%, P  = 0.15 respectively). There was no evidence for a change in FMD within either arm from baseline to week 12 or week 24, or between arms at any timepoint.(Table 2 & 3). We also did not find evidence between a difference between arms at any time point in brachial artery diameter or endothelial independent vasodilation (following administration of nitroglycerin, data not shown). Reactive hyperemia, an assessment of microvascular dysfunction, appeared to improve in the mesalamine to placebo arm from week 12 to week 24 (P<0.01), but this was not significantly different in comparison to the placebo to mesalamine arm during the same time period (P  = 0.09).

### Adverse events

Overall, the study medication was well tolerated. One subject who was randomized to mesalamine declined participation before the first follow-up visit. Another subject had underlying liver cirrhosis and ascites, and was excluded from the study. Two subjects died in the study, one in the placebo arm was assaulted and had an intracranial hemorrhage, and another had a sudden death. Regarding the individual with the sudden death, routine labs including creatinine and a complete blood count were stable two weeks earlier, and the impression of the data safety and monitoring board was that the sudden death was likely to be unrelated to mesalamine. One subject with a drug-dependency relapsed and dropped out of the study along with another two who were unable to commit to the requirements of the study.

## Discussion

Observational studies and theoretical considerations have suggested that targeting mucosal inflammation during chronic HIV infection might improve intestinal barrier function, reduce MT, and thereby have beneficial immunologic effects. At least under the conditions studied here, this randomized trial showed no evidence for such effects by mesalamine, a mucosally active anti-inflammatory agent used clinically to decrease mucosal inflammation in the setting of inflammatory bowel disease.

While the mechanism of action of mesalamine is not well understood, it remains clinically effective in mild to moderate ulcerative colitis, possibly because it can modulate several inflammatory pathways (including those associated with PPARγ, arachidonic acid and leukotriene biosynthesis, NFκB, and mTOR). [42], [43], [44], [45], [46], [47] While pill counts suggested high adherence, it is possible that suboptimal adherence remained a problem since drug levels were not monitored. Given the absence of an observed effect of mesalamine, these pathways may not be critical to the immune activation and MT associated with HIV disease. Alternatively, the particular formulation of mesalamine used in this study, which is targeted to release in the terminal ileum and below, might not have been effective in decreasing mucosal inflammation and MT in the upper gastrointestinal tract. We chose this particular formulation since the microbial density is much higher in the lower than the upper gastrointestinal tract, but it remains plausible that a significant amount of MT occurs above the terminal ileum in HIV infection. Nevertheless, we failed to find any evidence of an effect of mesalamine even on rectal T cell activation, where the drug should have concentrated, suggesting that mesalamine is unlikely to affect the key pathways driving immune activation and gut barrier defects at this mucosal location.

The possibility that an anti-inflammatory agent might ameliorate HIV-associated inflammation has been supported by studies of COX-2 inhibitors. HIV gp120 has been shown to induce COX-2. [48], [49], [50] Targeted therapy with specific COX-2 inhibitors has been reported to reduce persistent immune activation, enhance perforin expression, and even augment vaccine responses. [23], [24], [25] Although inhibition of COX-2 and lipoxygenase pathways by mesalamine has been reported, [51], [52] targeting of COX-2 by mesalamine is likely modest compared to COX-specific inhibitors. Even though there have been promising results with COX-2 inhibitors, the association of these agents with cardiovascular events limits their use. A pilot trial of aspirin has also shown promise [53] and is being followed up with a randomized controlled trial in the AIDS Clinical Trial Group.

Several other approaches to decreasing MT have been assessed recently in clinical trials, with mostly disappointing results. For example, sevelamer (a locally active chelator of LPS, phosphate, and lipid), was found to decrease serum LDL levels in untreated HIV infection; yet, it had no effect on other markers of systemic MT. [54] Similarly, the luminally active antibiotic, rifaximin, while modestly reducing systemic T cell activation, failed to decrease any specific markers of MT. [55] Other recent trials of pre- and pro-biotics have also had equivocal effects on systemic markers, but without consistent effects on specific markers of MT. [56], [57] Multiple pathways in addition to MT contribute to the persistent inflammatory state observed in ART-suppressed HIV-infected individuals (e.g., ongoing viral replication [58], co-infections [59], and defective phagocytosis [9]) and may be a reason we did not observe an effect of mesalamine.

Overlapping serologic markers against bacterial antigens in both IBD and HIV suggest evidence for MT in each condition. [21] Two-thirds of patients with advanced HIV disease exhibit markers often used to identify patients with IBD [e.g., antibodies to Saccharyomyces cerevesiae (ASCA), outer membrane to porin C of E. Coli (Omp-C), bacterial flagellin (CBir1), and pANCA]. [60] However, the absence of an effect of mesalamine in treated HIV infection suggests that different mechanisms may underlie the translocation of microbes in each condition. One difference is that HIV eliminates activated CD4+ T cells, particularly the Th17 and Th22 subsets, cells critical to the maintenance of mucosal immunity and epithelial barrier function. [61], [62] By contrast, IBD has been linked to an exuberant innate response to microbes through innate sensors such as nucleotide oligomerization domain 2 (NOD2), autophagy, and components of the Th17 pathway. [63] Perhaps these fundamental differences account for the relative lack of efficacy of mesalamine in the context of HIV disease.

In summary, we found that the addition of mesalamine to suppressive ART did not reduce immune activation, biomarkers of inflammation, and low-level viremia in the peripheral blood, nor did we observe an effect of mesalamine in the rectal mucosa or on endothelial function. Since MT and immune activation remain an important contributor to morbidity and mortality in this population, alternative strategies to reduce inflammation are warranted.

## Supporting Information

S1 CONSORT Checklist CONSORT 2010 checklist of information to include when reporting a randomised trial.
(DOC)

S1 Dataset Mesalamine dataset for public use.
(XML)

S1 Protocol Protocol approved by the UCSF Committee on Human Research.
(PDF)
