# Supplementary material for: The Immunologic Effects of Mesalamine in Treated HIV-Infected Individuals with Incomplete CD4+ T Cell Recovery: A Randomized Crossover Trial
Source: PLoS One. 2014 Dec 29;9(12):e116306. doi: 10.1371/journal.pone.0116306 (PMC4283685; doi:10.1371/journal.pone.0116306)
Supplement: S1 Protocol — Protocol approved by the UCSF Committee on Human Research. (PDF) [file pone.0116306.s003.pdf]

Appendix S1. Protocol approved by the UCSF Committee on Human Research

9.4 Check any research programs this study is associated with:

- □ Cancer Center
- □ Center for AIDS Prevention Sciences (CAPS)
- □ Global Health Sciences
- □ Immune Tolerance Network (ITN)
- □ Osher Center
- ■ Positive Health Program

10.0 Study Design

10.1 * Study design:

While most HIV-infected patients can now achieve nearly complete viral suppression on currently available HIV medications, they still have at least a 10-year shorter life expectancy than the general population and are at higher risk for diseases associated with accelerated aging including cardiovascular disease and non-AIDS-defining cancers. Persistent inflammation and immune activation are believed to drive this increased risk. Despite suppression of viral replication in peripheral blood by effective HIV medications, HIV may continue to be expressed at low levels by T cells in the lining of the gut and may also result in translocation of bacterial products across the lining of the gut, driving persistent inflammation. We believe that decreasing inflammation directly in the gut may decrease both of these potential causes of chronic inflammation, potentially resulting in an immunologic benefit. Mesalamine is an oral anti-inflammatory drug used to treat patients with inflammatory bowel disease, acts locally on the gut tissue to decrease inflammation, and is associated with very few side effects. In this study, we will test whether 12 weeks of mesalamine therapy decreases systemic immune activation and inflammation in HIV-infected patients with viral suppression on HIV medications. If mesalamine therapy reduces immune activation and inflammation in our study, it would prompt larger studies to see if mesalamine decreases clinical outcomes like cardiovascular disease, cancer, and mortality in this setting.

10.2 Check all that apply:

- □ Phase I
- □ Phase II
- □ Phase III
- ■ Phase IV

11.0 Scientific Considerations

11.1 Hypothesis:

This study has a hypothesis:
● Yes ○ No

If yes, state the hypothesis or hypotheses:

Hypothesis 1: Compared to those randomized to early placebo therapy, subjects randomized to early mesalamine therapy will experience greater reductions in the frequency of activated CD8+ T cells and plasma levels of biomarkers of microbial translocation (i.e., LPS, sCD14, 16S rRNA levels) at week 12.

Hypothesis 2: As a consequence of interrupting the vicious cycle of gut-associated lymphoid tissue (GALT) HIV RNA release, microbial translocation, and Th17 depletion, subjects randomized to early mesalamine will have sustained reductions in systemic T cell activation and microbial translocation even after crossing over into the placebo arm at week 12.

11.2 * List the specific aims:

To assess whether 12 weeks of oral mesalamine therapy decreases the frequency of circulating activated (CD38+ HLA-DR+) CD8+ T cells and systemic markers of microbial translocation in HIV-infected individuals maintaining treatment-mediated viral suppression.

11.3 Statistical analysis:

The primary outcome will be the reduction in the % activated (CD38+ HLA-DR+) CD8+ T cells in peripheral blood at week 12 compared between treatment arms. We hypothesize that mesalamine-treated subjects will have a greater reduction in CD8+ T cell activation at week 12 than placebo-treated subjects. The week 12 change in CD8+ T cell activation will be compared between groups with a two-tailed T test if normally distributed or a Wilcoxon ranksum test if non-normally distributed. We will also use generalized estimating equations with timepoint-treatment arm interaction terms to assess changes in CD8+ T cell activation over time using all available timepoints. Outcome variables will be transformed if necessary to satisfy model assumptions. Model residuals and linear predictions will be assessed to ensure that model assumptions are satisfied and the effects of influential outliers will be investigated and reported for all models.

In secondary analyses, we also hypothesize that mesalamine-treated subjects will experience greater week 12 decreases in IDO activity, microbial translocation, and inflammatory biomarkers than placebo-treated subjects, but greater week 12 increases in the Th-17/Treg ratio. We also hypothesize that reductions in CD8+ T cell activation will persist after mesalamine-treated subjects cross over to the placebo arm at week 12 as a consequence of interrupting the vicious cycle of GALT inflammation, HIV RNA release, Th-17 depletion, and microbial translocation. These within-group changes in CD8+ T cell activation and all other inflammatory mediators will be assessed with generalized estimating equations. In additional secondary analyses, we will assess within-group changes in all of the above parameters during the first 12 weeks of mesalamine therapy using generalized estimating equations and combining both those randomized to early mesalamine therapy and

those initiating mesalamine at week 12.

11.4 * **This is an investigator-initiated study:**

● Yes ○ No

11.5 **This study has received scientific or scholarly review from (check all that apply):**

- ☐ Cancer Center Protocol Review Committee (PRC) (Full approval or contingent PRC approval is required prior to final CHR approval for cancer-related protocols.)
- ☐ CTSI Clinical Research Center (CRC) advisory committee
- ☐ Departmental scientific review
- ☐ Other:

Specify **Other**:

If applicable, attach the Departmental Scientific Review Form at the end of the application.

12.0 Background

12.1 **Background:**

Background

While most HIV-infected individuals are now able to achieve and maintain viral suppression on modern antiretroviral therapy (ART) regimens, they remain at higher risk than the general population for a variety of non-AIDS-associated diseases including cardiovascular disease, liver disease, renal disease, and non-AIDS associated malignancies[1, 2]. Several studies have suggested that persistently low CD4+ T cell counts despite suppression increase the risk of these non-AIDS-associated conditions[3-5]. Persistent inflammation and immune activation has been well documented in this population and have been shown to predict both poor CD4+ T cell recovery and earlier mortality in this setting. Since persistent inflammation and immune activation has emerged as a major mediator of this increased risk of morbidity and mortality, interventions designed to decrease immune activation are urgently needed. Our proposed study targets two of the leading potential determinants of persistent immune activation in this setting: persistent viral release in gut-associated lymphoid tissue and microbial translocation. Thus, our study addresses one of the most important clinical problems for HIV-infected individuals and also two of the most important features of HIV pathogenesis. If we find that mesalamine decreases T cell activation in HIV-infected individuals, it will lead to much larger multi-center studies to determine whether this or related interventions designed to decrease inflammation in gut-associated lymphoid tissue decrease the incidence of premature mortality and morbidity from diseases associated with accelerated aging (serious bacterial infections, non-AIDS-associated cancer, cardiovascular disease, stroke, renal disease, liver disease,

osteoporosis, etc.). Achieving a better understanding of the mechanisms responsible for persistent microbial translocation despite treatment-mediated viral suppression should also pave the way for identifying novel interventions other than mesalamine.

HIV-infected individuals have an abnormally short life expectancy despite modern ART. Most HIV-infected individuals with access to antiretroviral therapy now achieve and maintain viral suppression. This has led to dramatically lower rates of AIDS-defining illnesses and marked improvements in life expectancy since the

pre-ART era. However, HIV-infected individuals continue to have at least a 10-year shorter life expectancy than the general population in the modern HAART era [6-10], and initiating ART at a low CD4+ T cell nadir has emerged as a consistent predictor of earlier mortality in this setting [9].

HIV-infected individuals are at high risk for non-AIDS-associated diseases typically associated with aging. As the incidence of AIDS-associated illnesses has declined dramatically in the modern HAART era, nearly 2/3 of all deaths among HIV-infected patients are now due to non-AIDS associated causes including malignancies, cardiovascular disease, renal disease, and liver disease [11]. HIV-infected individuals remain at much higher risk than the general population for a variety of non-AIDS-associated cancers including Hodgkin lymphoma, anal, lung, liver, oropharyngeal, and colorectal cancers, many with incidence rates that are actually increasing in the modern HAART era [2]. Similarly, while cardiovascular disease event rates may be declining in recent years as a consequence of improved management of traditional risk factors [12], several recent large cohort studies have reported that HIV-infected individuals have a 1.5- to 2.0-fold higher risk of cardiovascular disease events than the general population after adjustment for traditional cardiovascular risk factors [13, 14]. HIV-infected individuals receiving antiretroviral therapy also remain at much higher risk of hospitalization for community-acquired pneumonia [15].

Why are HIV-infected patients at high risk for non-AIDS-associated morbidity/mortality? Several lines of evidence suggest that HIV itself may increase the risk for non-AIDS-associated morbidity independent of health-related behaviors and drug toxicity. For example, in the SMART trial, HIV-infected individuals randomized to continuous antiretroviral therapy had fewer non-AIDS-associated events than those randomized to intermittent therapy guided by CD4+ T cell counts [16]. Furthermore, persistently low CD4+ T cell counts during antiretroviral therapy have been consistently associated with an increased risk of non-AIDS-associated morbidities [3, 5, 15, 17]. However, persistent CD4+ lymphopenia cannot be the sole factor explaining the increased risk of non-AIDS-associated morbidities in HIV-infected individuals. The incidence of many non-AIDS-associated cancers is rising despite increasing CD4+ T cell counts in the HIV-infected population [2], and HIV-infected individuals restoring normal CD4+ T cell counts during

antiretroviral therapy continue to have a 6-fold greater risk of hospitalization for pneumonia than the general population [15]. Furthermore, the ESPRIT and SICLAAT trials recently confirmed that while IL-2 administration significantly increases CD4+ T cell counts, it has no impact on clinical outcomes in HIV-infected individuals receiving HAART [18, 19].

Potential role of inflammation in driving morbidity/mortality in treated HIV infection. We and others have characterized the persistent immunologic perturbations in HIV-infected individuals maintaining treatment-mediated viral suppression to identify targets for future interventions. For example, despite marked reductions during early antiretroviral therapy, T cell activation levels remain abnormally high despite years of treatment-mediated viral suppression and higher T cell activation levels have been associated with blunted CD4+ T cell recovery [20-23]. Similarly, plasma biomarkers associated with inflammation (IL-6) and coagulation (d-Dimer) remain abnormally elevated in treated HIV infection and have been associated with earlier mortality and cardiovascular events [24]. While the specific causes of persistent inflammation and immune activation among HIV-infected individuals maintaining treatment-mediated viral suppression have not been definitively established, residual low-level HIV RNA in lymphoid tissues or plasma [25, 26], and microbial translocation [27, 28], all likely play an important role. We hypothesize that by decreasing gut-associated lymphoid tissue (GALT) inflammation with mesalamine in our proposed trial, we will decrease HIV expression from latently infected cells in GALT and decrease microbial translocation, both leading to lower systemic immune activation levels.

Mesalamine decreases GALT inflammation in patients with inflammatory bowel disease. Mesalamine (5-aminosalicylic acid) is now available in a once-per-day dosing regimen and is commonly used for the treatment of mild to moderately active ulcerative colitis. Mesalamine is a cylooxygenase inhibitor and preferentially decreases prostaglandin production in the colon, reduces GALT inflammation, and both induces and maintains remission of ulcerative colitis in HIV-uninfected individuals within 8 weeks [29-32]. Systemic administration of cyclooxygenase inhibitors has also recently been shown to decrease inflammation in the lymphoid tissues of untreated HIV-infected individuals [33]. However, the toxicities associated with chronic systemic NSAID use (i.e., renal toxicity, liver toxicity, and gastrointestinal bleeding) limit their widespread use in HIV-infected individuals. Since mesalamine is only ~25% systemically bio-available, it acts primarily within the GALT and is extremely well tolerated, with fewer patients discontinuing mesalamine than placebo in clinical trials. We hypothesize that mesalamine will decrease both GALT inflammation in HIV-infected individuals, blocking the vicious cycle of local HIV RNA release, Th17 depletion, and microbial translocation, and resulting in decreased systemic T cell activation levels.

Significance

This proposal is a novel treatment strategy for HIV infection. Now that the vast majority of HIV-infected individuals are able to achieve and maintain viral suppression on modern antiretroviral therapy regimens, the primary need – likely for the next decade - is for new interventions to decrease persistent inflammation and immune activation, which are believed to be primary mediators of premature mortality and morbidities associated with accelerated aging in this setting. This proposal tests an innovative approach to decrease immune activation in this setting and flows directly from cutting edge concepts in HIV pathogenesis. If we find that mesalamine decreases T cell activation in HIV-infected individuals, it will lead to much larger multi-center studies to determine whether this or related interventions designed to decrease inflammation in gut-associated lymphoid tissue decrease the incidence of premature mortality and morbidity from diseases associated with accelerated aging (serious bacterial infections, non-AIDS-associated cancer, cardiovascular disease, stroke, renal disease, liver disease, osteoporosis, etc.). Achieving a better understanding of the mechanisms responsible for persistent microbial translocation despite treatment-mediated viral suppression should also pave the way for identifying novel interventions other than mesalamine.

12.2 Preliminary studies:

Abnormal T cell activation persists despite treatment-mediated viral suppression and is associated with blunted CD4+ T cell recovery. We hypothesized that abnormal T cell activation levels might persist to varying degrees despite ART-mediated viral suppression and limit the extent of CD4+ T cell recovery. To address this issue, we performed a cross-sectional analysis of T cell activation levels in 99 ART-treated patients maintaining plasma HIV RNA levels ≤1,000 copies/ml, 13 untreated patients, and 6 uninfected participants [34]. Despite a median of 21 months of viral suppression, the HIV-infected patients had higher levels of activated (CD38+HLA-DR+) CD4+ T cells and CD8+ T cells than uninfected controls ($P<0.001$ for both), even when restricting the analysis to those with persistently undetectable plasma HIV RNA levels and those without hepatitis C virus co-infection. Furthermore, higher T cell activation was associated with fewer CD4+ T cell gains during therapy ($p<0.001$). These data support our hypothesis that abnormal immune activation persists despite long-term suppressive ART and may contribute to persistent immune defects. We will assess whether mesalamine therapy reduces systemic T cell activation in this setting in Aim 1.

Microbial translocation is associated with T cell activation in HIV-infected patients with undetectable viremia. Recently, we and others have sought to characterize the determinants of persistent T cell activation among HIV-infected patients with clinically undetectable viremia in ART. Since microbial translocation may contribute to T cell activation in untreated HIV infection [35], we hypothesized that microbial translocation might persist and continue to drive T cell activation even after long-term suppression of plasma HIV RNA levels. To address this, we measured plasma lipopolysaccharide (LPS) and bacterial 16S rDNA levels in HIV-infected patients maintaining plasma HIV RNA levels <75 copies/ml for a median of 28 months (n=114)

and HIV-uninfected individuals (n=31) [27, 36]. The ART-suppressed participants had higher plasma LPS levels than HIV-uninfected individuals (P<0.001). Furthermore, among ART-treated patients, higher plasma 16S rDNA levels were associated with higher % activated (CD38+HLA-DR+) CD8+ T cells (rho: 0.19, P=0.047) and fewer treatment-mediated CD4+ T cell gains (rho: -0.22, P=0.02). Our findings of persistent microbial translocation despite ART have also been recently confirmed by others [28]. We will assess whether mesalamine therapy reduces persistent microbial translocation in Aim 1.

Th-17 depletion may contribute to microbial translocation and immune activation in SIV and HIV infections. Th-17 cells are a recently defined (typically CD4+) T cell subset that responds to bacterial and fungal pathogens and are thought to play a role in clearing translocated microbial products and in maintaining the integrity of the gut epithelial mucosal barrier, thereby decreasing systemic microbial translocation [37-39]. Our group and others have recently established that these cells are preferentially depleted from GALT early in pathogenic SIV and HIV infections, yet preserved in non-pathogenic SIV infection of African green monkeys and sooty mangabeys [37-39]. In addition to this early and sustained depletion of Th-17 cells in GALT, our group has demonstrated a concurrent and progressive expansion of regulatory CD4+ T cells (Tregs) during progressive pathogenic SIV infection, presumably as a consequence of chronic immune activation [38]. In this SIV model, lower Th-17/Treg ratios were strongly associated with higher systemic T cell activation levels (P=0.03). Interestingly, Th-17 and Tregs share a common progenitor, so our group has hypothesized that the chronic inflammatory microenvironment in GALT among HIV-infected individuals may result in shunting of progenitors down the Treg pathway, blocking the restoration of Th-17 cells in GALT.

In work recently submitted for publication, we assessed Th-17/Treg ratios in both peripheral blood and GALT in untreated HIV-infected controllers (VL<2000 c/ml) and non-controllers (VL>10,000 c/ml), as well as ART-suppressed patients and HIV-negative controls. Compared to HIV-negative individuals, ART-suppressed patients continued to have significantly lower Th17/Treg ratios (Figure A, P<0.005). Furthermore, among untreated HIV-infected controllers and non-controllers, lower Th-17/Treg ratios in GALT were strongly associated with higher frequencies of activated (CD38+) CD8+ T cells in GALT (Figure B, P=0.0008). In further *in vitro* experiments, induction of indole-2,3-dioxygenase (IDO) by inflammatory cytokines including interferon-γ resulted in Th-17 cell depletion *in vitro*. Higher IDO mRNA expression in GALT in untreated HIV-infected individuals was also associated with decreased GALT Th-17/Treg ratios (rho: -0.69, P<0.001). These results suggest that Th17/Treg imbalance persists during treatment-mediated viral suppression and may be both a cause and a consequence of a persistent inflammatory microenvironment in GALT.

12.3 References:

[illegible]spoon, S.K., et al., *State of the science conference: Initiative to decrease cardiovascular risk and [illegible]ease quality of care for patients living with HIV/AIDS: executive summary*. Circulation, 2008. **11**[illegible] [illegible]210.

[illegible], P., et al., *Incidence of types of cancer among HIV-infected persons compared with the general [illegible]lation in the United States, 1992-2003*. Ann Intern Med, 2008. **148**(10): p. 728-36.

[illegible]er, J.V., et al., *CD4+ count and risk of non-AIDS diseases following initial treatment for HIV inf*[illegible]

, 2008. **22**(7): p. 841-8.

n, D., et al., *Current CD4 cell count and the short-term risk of AIDS and death before the availa[illegible] tive antiretroviral therapy in HIV-infected children and adults.* J Infect Dis, 2008. **197**(3): p. 39[illegible]

forte, A., et al., *HIV-induced immunodeficiency and mortality from AIDS-defining and non-AID[illegible] ing malignancies.* Aids, 2008. **22**(16): p. 2143-53.

e, N., et al., *Survival of persons with and without HIV infection in Denmark, 1995-2005.* Ann In[illegible] , 2007. **146**(2): p. 87-95.

den, C., et al., *HIV-Infected Adults With a CD4 Cell Count Greater Than 500 Cells/mm3on Long[illegible] bination Antiretroviral Therapy Reach Same Mortality Rates as the General Population.* J Acqu[illegible] une Defic Syndr, 2007.

, V.D., et al., *Continued improvement in survival among HIV-infected individuals with newer fo[illegible] ly active antiretroviral therapy.* Aids, 2007. **21**(6): p. 685-92.

aboration, T.A.T.C., *Life expectancy of individuals on combination antiretroviral therapy in hig[illegible] ries: a collaborative analysis of 14 cohort studies.* The Lancet, 2008. **372**(9635): p. 293-299.

karan, K., et al., *Changes in the risk of death after HIV seroconversion compared with mortality [illegible] ral population.* Jama, 2008. **300**(1): p. 51.

den, C., et al., *Changes in causes of death among adults infected by HIV between 2000 and 2005[illegible] rtalite 2000 and 2005" surveys (ANRS EN19 and Mortavic).* J Acquir Immune Defic Syndr, 200[illegible] 0-8.

ey, L., et al. *Updated surveillance of cardiovascular event rates among HIV-infected and HIV-fected Californians, 1996 to 2008*. in *in Program and Abstracts from the 16th Conference on oviruses and Opportunistic Infections, 2009, Montreal, CA, Abstract #710*. 2009.

nt, V.A., et al., *Increased acute myocardial infarction rates and cardiovascular risk factors amo[illegible] nts with human immunodeficiency virus disease.* J Clin Endocrinol Metab, 2007. **92**(7): p. 2506-[illegible]

ier, J.S., et al., *Coronary heart disease in HIV-infected individuals.* J Acquir Immune Defic Syn[illegible]): p. 506-12.

ard, O.S., et al., *Hospitalization for pneumonia among individuals with and without HIV infecti[illegible] -2007: a Danish population-based, nationwide cohort study.* Clin Infect Dis, 2008. **47**(10): p. 1[illegible]

dr, W.M., et al., *CD4+ count-guided interruption of antiretroviral treatment.* N Engl J Med, 20[illegible] 22): p. 2283-96.

n, B., et al. *Association between CD4 and HIV RNA with non AIDS-related causes of death in th[illegible]*

bination antiretroviral therapy (cART)* in *4th IAS Conference on HIV Pathogenesis, Treatment, a
ention. July 22-25, 2007. Sydney. Abstract WEPEB019*. 2007.

, Y. and S.S. Committee. *Effect of interleukin-2 on clinical outcomes in patients with CD4+ cel
299/mm3: Primary results of the SILCAAT study*. in *In the Program and Abstracts of the 16th
erence on Retroviruses and Opportunistic Infections, Montreal, CA, 2009: Abstract #90bLB.*

o, M., D. Abrams, and I.E.S. Group. *Effect of interleukin-2 on clinical outcomes in patients with
+ cell count of 300/mm3: Primary results of the ESPRIT study*. in *In the Program and Abstracts
Conference on Retroviruses and Opportunistic Infections, Montreal, CA, 2009: Abstract #90aL*

t, P.W., et al., *T Cell Activation Is Associated with Lower CD4+ T Cell Gains in Human
unodeficiency Virus-Infected Patients with Sustained Viral Suppression during Antiretroviral Th*
t Dis, 2003. **187**(10): p. 1534-43.

dhi, R.T., et al., *Effect of baseline- and treatment-related factors on immunologic recovery after
ation of antiretroviral therapy in HIV-1-positive subjects: results from ACTG 384.* J Acquir Imm
Syndr, 2006. **42**(4): p. 426-34.

lez, H., et al., *Limited immune restoration after 3 years' suppression of HIV-1 replication in pati
erately advanced disease.* AIDS, 2002. **16**(14): p. 1859-1866.

oechea, M., et al., *Determinants of CD4+ T cell recovery during suppressive antiretroviral ther
ciation of immune activation, T cell maturation markers, and cellular HIV-1 DNA.* J Infect Dis,
1): p. 29-37.

er, L.H., et al., *Inflammatory and coagulation biomarkers and mortality in patients with HIV inf*
Med, 2008. **5**(10): p. e203.

n, P.A., et al., *Multiple measures of HIV burden in blood and tissue are correlated with each ot
vith clinical parameters in aviremic subjects.* AIDS, 2003. **17**(1): p. 53-63.

larelli, F., et al., *ART suppresses plasma HIV-1 RNA to a stable set point predicted by pretherap
nia.* PLoS Pathog, 2007. **3**(4): p. e46.

g, W., et al., *Plasma Levels of Bacterial DNA Correlate with Immune Activation and the Magnit
une Restoration in Persons with Antiretroviral-Treated HIV Infection.* J Infect Dis, 2009.

chetti, G., et al., *Microbial translocation is associated with sustained failure in CD4+ T-cell
nstitution in HIV-infected patients on long-term highly active antiretroviral therapy.* Aids, 2008.
35-8.

m, M.A., et al., *Once-daily, high-concentration MMX mesalamine in active ulcerative colitis.*
roenterology, 2007. **132**(1): p. 66-75; quiz 432-3.

tenstein, G.R., et al., *Effect of once- or twice-daily MMX mesalamine (SPD476) for the inductio[illegible] ssion of mild to moderately active ulcerative colitis.* Clin Gastroenterol Hepatol, 2007. **5**(1): p. 9[illegible]

erland, L. and J.K. Macdonald, *Oral 5-aminosalicylic acid for maintenance of remission in ulce[illegible] is.* Cochrane Database Syst Rev, 2006(2): p. CD000544.

erland, L. and J.K. Macdonald, *Oral 5-aminosalicylic acid for induction of remission in ulcerati[illegible] is.* Cochrane Database Syst Rev, 2006(2): p. CD000543.

rouw, N., et al. *A 14-day treatment with a cyclooxygenase 2 (COX-2) inhibitor reduces inflamm[illegible] ymphoid tissues of HIV-infected patients without HAART - a pilot study with positron emission graphy (PET/CT) assessment* in *In the Program and Abstracts of the 5th IAS Conference on HI[illegible] ogenesis, Treatment, and Prevention, Cape Town, South Africa, Abstract # MOAB206*. 2009.

t, P.W., et al., *T Cell Activation Is Associated with Lower CD4+ T Cell Gains in Human unodeficiency Virus-Infected Patients with Sustained Viral Suppression during Antiretroviral Th[illegible]* t Dis, 2003. **187**(10): p. 1534-43.

chley, J.M., et al., *Microbial translocation is a cause of systemic immune activation in chronic [illegible] tion.* Nat Med, 2006.

t, P.W., et al., *Relationship between T Cell Activation and CD4(+) T Cell Count in HIV-Seropos[illegible] iduals with Undetectable Plasma HIV RNA Levels in the Absence of Therapy.* J Infect Dis, 2008 1): p. 126-133.

chley, J.M., et al., *Differential Th17 CD4 T cell depletion in pathogenic and nonpathogenic lent[illegible] tions.* Blood, 2008.

e, D., et al., *Critical loss of the balance between Th17 and T regulatory cell populations in path[illegible] infection.* PLoS Pathog, 2009. **5**(2): p. e1000295.

r, M., et al., *Alpha4(+)beta7(hi)CD4(+) memory T cells harbor most Th-17 cells and are prefer[illegible] ted during acute SIV infection.* Mucosal Immunol, 2009. **2**(5): p. 439-49.

igan-O'Connor, D.J., K. Abel, and J.M. McCune, *Suppression of SIV-specific CD4+ T cells by i[illegible] dult macaque regulatory T cells: implications for SIV disease progression.* J Exp Med, 2007. **20**[illegible] 79-92.

chley, J.M., et al., *Microbial translocation is a cause of systemic immune activation in chronic [illegible] tion.* Nat Med, 2006. **12**(12): p. 1365-71.

, W., et al., *Plasma levels of bacterial DNA correlate with immune activation and the magnitud[illegible] une restoration in antiretroviral-treated HIV infection.* J Infect Dis, 2009. **(in press)**.

hens, R.L. and P.A. Thompson, *Modulatory effects of sCD14 and LBP on LPS-host cell interact[illegible]*

toxin Res, 2005. **11**(4): p. 225-9.

If you have a separate bibliography, attach it to the submission with your other study documents.

13.0 Sample Size and Eligibility

13.1 Number of subjects that will be enrolled at UCSF and affiliated institutions (locally):

30

13.2 Total number of subjects that will be enrolled at all sites (for study overall):

n/a

13.3 Estimated number of people that you will need to consent and screen here (but not necessarily enroll) to get the needed subjects:

40

13.4 Sample size calculation:

Our primary hypothesis is that mesalamine-treated subjects will have greater week 12 decreases in CD8+ T cell activation than placebo-treated subjects. In our prior longitudinal studies of HIV-infected individuals receiving antiretroviral therapy, the standard deviation of the week 12 change in the % activated CD8+ T cells was 3.3%. Assuming a standard deviation as high as 4% and a 5% Type I error rate, we would have 80% power to detect a difference as small as 4.4% in the week 12 change in activated CD8+ T cells between groups if we enrolled 13 subjects in each arm. This is a comparable effect size to that we observed in our recent trial of valganciclovir (difference of 4.5% between groups at week 8). We plan to enroll 15 subjects in each arm to account for premature treatment discontinuations.

13.5 * Eligible age range(s):

- ☐ 0-6 years
- ☐ 7-12 years
- ☐ 13-17 years
- ■ 18+ years

13.6 Inclusion criteria:

1. HIV-1 infection, as documented by any licensed ELISA test kit and confirmed by Western blot at any time prior to study entry.

2. Stable antiretroviral therapy for at least 6 months

3. Screening CD4+ T cell count below 350 cells/mm^3

4. All available CD4+ T cell counts in the last year and at screening <350 cells/mm^3

5. Screening plasma HIV RNA levels below level of detection (< 40 copies RNA/mL).

6. All available plasma HIV RNA levels within past year below the level of detection. Isolated detectable values < 500 c/ml are allowed if HIV RNA levels before and after this time point are undetectable.

7. >90% adherence to therapy within the preceding 30 days, as determined by self-report.

8. Both male and female subjects are eligible. Females of childbearing potential must have negative pregnancy test at screening and agree to use a double-barrier method of contraception during the study.

13.7 Exclusion criteria:

1. Patients who are intending to modify antiretroviral therapy in the next 24 weeks for any reason.

2. Serious illness requiring hospitalization or parental antibiotics within preceding 3 months.

3. Exposure to any immunomodulatory drug in the past 16 weeks.

4. Active hepatitis C or hepatitis B which will require treatment in the subsequent 24 weeks.

5. Screening absolute neutrophil count <1,000 cells/mm^3, platelet count <50,000 cells/mm^3, Hgb < 8mg/dL

6. Acute pancreatitis.

7. Renal insufficiency with creatinine clearance less than 50 ml/min

8. Elevated transaminases greater than 2.5 times the upper limit of normal.

9. Evidence of decompensated cirrhosis, heart failure.

10. Pregnant or breastfeeding women

13.8	There are inclusion or exclusion criteria based on gender, race or ethnicity:

○ Yes ● No

If **yes**, please explain the nature and rationale for the restrictions:

14.0 Drugs and Devices

14.1 * Drugs or biologics will be studied under this application:

● Yes ○ No

14.2 * Investigational medical devices or in vitro diagnostics will be used OR approved medical devices or in vitro diagnostics will be studied under this application:

○ Yes ● No

14.3 * A Non-Significant Risk (NSR) determination is being requested for an investigational device:

○ Yes ● No

14.4 Verification of IND/IDE numbers: If the sponsor's protocol does not list the IND/IDE number, you must submit documentation from the sponsor or FDA identifying the IND/IDE number for this study. Attach this documentation in the Other Study Documents section of the Initial Review Submission Packet.

15.0 Study Drug Details

15.1 List the drugs or biologics that will be studied:

View Details	Drug Name		FDA Approved	A new drug or a new use of approved drug:	IND Number
	**Trade Drug Name:**	APRISO	Yes	No	108315
	**Generic Drug Name:**	MESALAMINE			
	**Investigational Drug Name:**				

Trade Drug Name:	APRISO
Generic Drug Name:	MESALAMINE
Investigational Drug Name:	
Identify the name of the manufacturer or source of investigational	Salix Pharmaceuticals, Inc.

drug/biologic:	
Is the drug supplied at no cost?	Yes
Is the Drug FDA Approved:	Yes
Is this a new drug or a new use of an already approved drug	No
Is an IND necessary	No
IND Number	108315
Who holds the IND:	PI holds the IND
IND details:	IND exemption number is 108315 and IND exemption letter is attached to this application.
If FDA Approved and an IND is not required, Please provide a rationale for exemption:	The investigation is not intended to be reported to FDA as a well-controlled study in support of a new indication for use, nor intended to be used to support any other significant change in the labeling for the drug.
Are you currently using this IND in another research project?	No
If yes, list the IRB Number(s):	
Will the investigational pharmacy be dispensing?	No
If the source is not a FDA licensed facility, provide details regarding the purity, quality, stability and sterility of the investigational drug/biologic:	

16.0 Other Approvals and Registrations

16.1 * This is a clinical trial:

● Yes ○ No

Clinical Trial Registration
"NCT" number for this trial:

NCT01090102

16.2 * Data from this study will be submitted to NIH for Genome-Wide Association Studies (GWAS):

○ Yes ● No

16.3 * This study involves vaccines produced using recombinant DNA technologies:

○ Yes ● No

16.4 * This study involves human gene transfer (NOTE: Requires NIH Recombinant DNA Advisory Committee (RAC) review prior to CHR approval):

○ Yes ● No

16.5 * The study protocol requires radiological procedures (e.g. CT scans, x-rays) or exposes subjects to radiation:

○ Yes ● No

16.6 This study involves other regulated materials and requires approval and/or authorization from the following regulatory committees:

□ Institutional Biological Safety Committee (IBC)

Specify BUA #:

□ Institutional Animal Care and Use Committee (IACUC)

Specify IACUC #:

□ Radiation Safety Committee

Specify RUA #:

□ Radioactive Drug Research Committee (RDRC)

Specify RDRC #:

□ Controlled Substances

17.0 Procedures

17.1 * List all study procedures, test and treatments required for this study:

Procedures

Randomization: Thirty subjects meeting inclusion criteria will be randomly assigned to oral mesalamine (1.5 gram/day) vs. matching placebo for the first 12 weeks, then crossed over into the opposite treatment arm for 12 weeks. Randomization will be in blocks of 2 to ensure an equal number of subjects in each treatment arm. Both investigators and subjects will be blinded to treatment assignment.

Detailed questionnaires and symptom assessments will be performed at each visit using validated teleforms, including detailed questions about self-reported adherence to both antiretroviral and study medications. These have been approved previously for the SCOPE study. Pill counts will also be performed at each visit.

Blood draws will be performed at each visit for clinical labs, real-time immunology assays, and plasma/PBMC cryopreservation. A medical staff will puncture the vein using a needle. Blood is usually taken from a vein on the back of the hand or just

below the elbow. Approximately 90 mL of blood will be drawn during each visit. Up to a total of 1200 mL (2 ¼ pints) will be drawn over an 8-month period, however, no more than 480 mL (2 cups) will be drawn over any 2-month period. There will be an optional consent for banking blood for future studies.

Stool collection will occur before randomization, before medication crossover, and at the end of the study. A bottle with preservative and a scooper will be provided to the patient. The day before an appointment, the patient will add a single scoop of stool into the sample bottle. The lid is screwed tightly and shaken for 30 seconds to mix stool and preservative. The sample is stored in the refrigerator and brought to the clinic the following day.

Measurements

Phenotyping for T cell activation (CD3, CD4, CD8, CD38, HLA-DR, and CCR5) will be performed on fresh whole blood and extracted mucosal mononuclear cells (MMC) in the McCune laboratory [38]. The primary outcome measure for T cell activation will be the %CD38+ HLA-DR+ CD8+ T cells.

Phenotyping for Tregs (CD25+ FoxP3+ CD127- CD4+ T cells) will be assessed by flow ctytometry on fresh whole blood and freshly extracted MMC using published protocols in the laboratory of Dr. McCune [40].

The % Th-17+ CD4+ T cells will also be measured on both fresh whole blood and MMC specimens in the McCune laboratory according to published protocols [38]. Cytokine assays will be performed *in vitro* on $5x10^5$ cells after no stimulation or stimulation with media, Staphylococcal enterotoxin B (1 µg/ml), or phorbol myristate acetate (PMA) (10 ng/ml) and ionomycin (1 µg/ml) for 6 hours at 37°C. Cytokine analysis will be performed after stringent gating of each cytokine positive population after PMA/ionomycin stimulation of $CD4^+$ or $CD8^+$ T cell populations from rectosigmoid cells or from PBMCs, and reported as background-subtracted values from the unstimulated cell population from each patient and for each stimulus. We also plan to assess the ratio of Th-17 to Treg cells in both peripheral blood and MMC.

Markers of microbial translocation (LPS and bacterial rDNA levels) will be measured on fasting plasma specimens in the McCune laboratory using kits and procedures we have used in the past [41, 42].

Inflammatory plasma biomarkers. Fasting plasma will be sent to the McCune laboratory for measurement of the following inflammatory biomarkers using commercially available ELISA kits: IL-6 (R&D Systems, Minneapolis, MN), d-Dimers (Asserachrom, Diagnostica Stago, France), high-sensitivity C-reactive protein (UBI Magiwel, United Biotech, Inc, Mountainview, CA), soluble TNF receptors (Biosource, Camarillo, CA), and soluble CD14 (R&D Systems, Minneapolis, MN). Soluble CD14 is of particular interest since it binds to and reflects stimulation of monocytes by

lipopolysaccharide (LPS) *in vivo*,[43] and may be a more reliable marker of microbial translocation in treated individuals than LPS or 16S rDNA levels [35].

Bacterial community. Stool samples will be sent to the Lynch laboratory for microbial community profiling. Using 30 ng of total DNA as template in triplicate reactions, perform 16S rRNA Q-PCR (40 cycles) to quantify bacterial burden present using the primers 16SF 5'-ACTCCTACGGGAGGCAGCAG-3' and 16SR 5'-TTACCGCGGCTGCTGG-3', in a standard 40 cycle reaction, using an annealing temperature of 55 °C. In addition, a series of 12 PCR reactions per sample using 30 ng of total DNA as template will be performed a gradient of annealing temperatures (48-56 °C; to maximize diversity amplified), using the universal 16S rRNA primers 27F and 1492R. products from all 12 reactions wil be pooled, purified and quantified before being fragmented, biotin labeled and hybridized to the 16S rRNA PhyloChip (developed by collaborators at Lawrence Berkeley National Laboratory). This is a culture-independent method to profie the majority of bacteria present in a sample and has the additional benefit of being able to detect microbes that constitute as little as 0.01% of a polymicrobial population.

Schedule of Events

Assay	- 4 Wk	Wk 0	Wk 2	Wk 4	Wk 8	Wk 12	Wk 14	Wk 16	Wk 20	Wk 24
Consent, medical history, pregnancy test, vital signs, adherence.	**x**									
Clinical Labs (CD4, CD8, VL, CBC, Metabolic panel)	**x**	**X** (+VL)	**x**	**x**	**x**	**X** (+VL)	**x**	**x**	**x**	**X** (+VL)
Peripheral Blood T cell activation, Th-17, Tregs, MT		**x**		**x**	**x**	**x**		**x**	**x**	**x**
Cryopreservation of plasma, serum and PBMC		**x**		**x**	**x**	**x**		**x**	**x**	**x**

←--------------------------------------→←--→

Mesalamine vs. Placebo X-over to Placebo vs.Mesalamine

If you have a procedure table, attach it to the submission with your other study documents.

17.2 Interviews, questionnaires, and/or surveys will be administered or focus groups will be conducted:

● Yes ○ No

List any standard instruments used for this study:

SCOPE Baseline ARVs (ver 04/26/06) SCOPE Follow-up ARVs (ver 12/07/05)

SCOPE Baseline Drugs (ver 06/01/07) SCOPE Follow-up Drugs (ver 06/01/07)

SCOPE Baseline General (ver 06/01/07) SCOPE Follow-up General (ver 06/01/07)
Bowel Questionnaire (ver 03/18/10)

Attach any non-standard instruments at the end of the application.

17.3 Conduct of study procedures or tests off-site by non-UCSF personnel:

○ Yes ● No

If yes, explain:

17.4 Sharing of experimental research test results with subjects or their care providers:

○ Yes ● No

If yes, explain:

17.5 * Specimen collection for future research and/or specimen repository/bank administration:

● Yes ○ No

17.6 Time commitment (per visit and in total):

Subjects will come in for a total of 10 study visits. The screening visit will take about one hour, the other 9 study visits will take about 30 minutes per visit.

17.7 Locations:

Subjects will be seen at 4C (GCRC), San Francisco General Hospital.

17.8 Describe the resources in place to conduct this study in a way that assures protection of the rights and welfare of participants:

The study will be conducted in conjunction with SFGH Ward 86 policy/procedure to insure patients receive support for physical, psychological and social needs related to HIV diagnosis and care.

18.0 Specimen Collection for Future Research and/or Specimen Repository/Bank Administration

(Note: This section replaces the old "Human Biologic Specimen Collecting and/or Banking for Future Research" supplement form. Please do not attach the old form to this application.)

18.1 Specimens are (check all that apply):

- ☐ Surplus clinical specimens from a diagnostic or therapeutic procedure
- ■ Specimens collected for research purposes only
- ☐ Other

If Other, explain:

18.2 Types of specimens:

- ■ Blood
- ☐ Tissue (describe below):
- ☐ Existing/archival materials (name source below):
- ■ Other (describe below):

Describe and/or name source:
DNA/RNA

18.3 Consent will be obtained via:

- ☐ Separate specimen banking consent form
- ■ Specimen banking section within a main research study consent form
- ☐ Surgical consent form with tissue donation brochure

18.4 Specimens will ultimately be stored (check all that apply):

UCSF

- ☐ UCSF repository/bank being established under this protocol
- ■ Existing UCSF specimen repository/bank with CHR approval

Provide the name of the bank and CHR approval number (if not being banked at UCSF under this protocol):

UCSF AIDS Specimen Bank
CHR approval number: 10035669

Outside Entity

- ☐ Cooperative group bank
- ☐ NIH
- ☐ Other university
- ☐ Industry sponsor

□ Other

Specify to what institution, cooperative group or company specimens will be transferred:

18.5 Direct identifiers will be sent with specimens or shared with other researchers and/or outside entities:

○ Yes
● No
○ N/A - Specimens will not be shared with others

If **Yes**, which identifiers will be sent with specimens:

□ Name
□ Date of birth
□ Social Security number
□ Medical record number
□ Address
□ Phone number
□ Email address
□ Other dates (surgery date, clinic visit dates, etc.)

If **Yes**, provide a justification for sending direct identifiers with the specimens:

19.0 Alternatives

19.1 Study drug or treatment is available off-study:

○ Yes
● No
○ Not applicable

19.2 * Is there a standard of care (SOC) or usual care that would be offered to prospective subjects at UCSF (or the study site) if they did not participate:

○ Yes ● No

If yes, describe the SOC or usual care that patients would receive if they choose not to participate:

If prospective subjects choose not to enroll, they will continue with their regular clinical care.

19.3 Describe other alternatives to study participation that are available to prospective subjects:

Subjects may choose not to participate.

20.0 Risks and Benefits

20.1 * Risks and discomforts:

**Risks of mesalamine:** Overall, the oral mesalamine preparation to be used in this trial (5-aminosalicylic acid, Apriso) is very well tolerated. When the mesalamine formulation and dose to be used in our trial was studied in patients with ulcerative colitis, FEWER serious adverse events were noted in the mesalamine arm than placebo arm (1.1% vs. 6.1%). Potential adverse events that have been observed in other studies of mesalamine products in patients with inflammatory bowel disease include an acute intolerance syndrome in 3% (manifested by cramping, abdominal pain, bloody diarrhea, and occasionally fever, headache, and rash). Rare cases of renal impairment (minimal change disease, acute or chronic interstitial nephritis) and myocarditis/pericarditis have also been reported with other mesalamine products. Compared to placebo, mesalamine has also been associated with a higher risk of headache (5.6% vs. 0.6%) and flatulence (4% vs.2.8%). Concurrent use of NSAIDS (i.e., ibuprofen, naproxen, etc) may increase the risk of renal impairment on mesalamine. Mesalamine may also increase the risk of blood disorders when co-administered with azathioprine or 6-mercaptopurine.<br><br>**Phlebotomy** may cause some discomfort, bleeding or bruising where the needle enters the skin, and rarely, fainting or infection may occur. Up to a total of 1200 mL (2¼ pints) will be drawn over an 8-month period, however, no more than 480 mL (2 cups) will be drawn over any 2-month period. This is within Red Cross Guidelines (less than 500 mL every two months). Risks of blood collection include anemia (low blood counts). You will be checked for anemia at each study visit. If the investigators feel that you are at significant risk for anemia, the amount of blood collected will be reduced. If your hemoglobin falls below 9 g/dL or your hematocrit falls below 27%, you will have 5 mL (1 teaspoon) of blood drawn to check your hemoglobin and hematocrit. Other than the blood required to check your hemoglobin and hematocrit, you will not have more blood drawn until your hemoglobin rises above 9 g/dL or your hematocrit rises above 27%.<br><br>**Confidentiality**: There is always a small risk of loss of privacy when participating in a clinical research study.	
**20.2 Steps taken to minimize risks to subjects:**	
Given the potential risk of renal toxicity, we are excluding patients with creatinine clearance <50. Since the intervention has not been adequately studied in pregnant or breastfeeding women and children, we are also excluding these individuals as well.<br><br>All subjects will be followed for possible adverse events throughout their involvement in the study. Specifically at each interaction, study personnel will elicit subject input as to discomforts or adverse experiences while	

taking the medication. A metabolic panel, liver function tests, and a CBC with differential will be performed at each visit. CD4+ T cell counts will also be performed regularly. These data will be evaluated by a Safety Monitoring Committee (SMC). Subjects will be screened for toxicity at each study visit and monitored with a basic metabolic panel and complete blood count for signs of asymptomatic toxicity.

Adverse events (AE) will be graded according to the Division of AIDS Table for Grading the Severity of Adult and Pediatric Adverse Events, Version 1.0, December 2004, which can be found on the Division of AIDS Regulatory Compliance Center Web Site: **http://rcc.tech-res-intl.com/eae.htm**.

All patients experiencing evidence of acute mesalamine intolerance syndrome (manifested by cramping, abdominal pain, bloody diarrhea, and occasionally fever, headache, and rash soon after initiating therapy) shall have the intervention discontinued.

Study medications will also be discontinued for decline in creatinine clearance by 50% of the baseline value or for creatinine clearance <40 ml/min.

If hemoglobin falls below 9 g/dL or hematocrit falls below 27%, subjects will have all research blood tests other than the blood required to check hemoglobin and hematocrit discontinued until the hemoglobin rises above 9 g/dL or hematocrit rises above 27%.

Other adverse events will be handled according to the AIDS Clinical Trial Group (ACTG) grading scale:

Grade 1 or 2

Subjects who develop a Grade 1 or 2 AE or toxicity may continue study medication.

Grade 3

If the investigator has evidence that the AE has NOT been caused by the study medication, dosing may continue. Subjects who develop a Grade 3 AE or toxicity thought to be related to study medication may have study medication withheld, at the investigator's discretion. The subject will be reevaluated weekly until the AE returns to Grade ≤ 2 or baseline, at which time the study medication may be reintroduced, at the discretion of the site

investigator. If the same Grade 3 AE recurs the study medication will be permanently discontinued and will be followed weekly until resolution of the AE.

Grade 4

Subjects who develop a Grade 4 symptomatic AE or toxicity (except lipid elevations) will have study medication permanently discontinued. Subjects experiencing Grade 4 AEs requiring permanent discontinuation of all ARV drugs will be followed weekly until resolution of the AE and encouraged to complete the permanent treatment discontinuation and follow-up protocol study evaluations.

20.3 Benefits to subjects:

● Yes ○ No

If yes, describe:

All subjects will receive study drug at some period during the trial. The study drug may decrease mucosal inflammation thereby leading to decreases in bacterial translocation and systemic immune activation and inflammation. These benefits may be long lasting.

20.4 Benefits to society:

Knowledge gained from this study may help in the future treatment of HIV-infected individuals. If mesalamine therapy reduces immune activation and inflammation in our study, it would prompt larger studies to see if mesalamine decreases clinical outcomes like cardiovascular disease, cancer, and mortality in this setting.

20.5 Explain why the risks to subjects are reasonable:

The potential risk associated with mesalamine therapy is quite small compared to the potential benefit of this intervention on systemic immune activation and inflammation, processes which are thought to mediate the increased risk of morbidity and mortality in individuals with treated HIV infection.

21.0 Data and Safety Monitoring Plan

21.1 Describe the plan for monitoring data and safety:

We have developed an independent Data Monitoring Committee (DMC). The DMC is composed of 3 independent individuals from the scientific community selected by the principal investigator and co-principal investigator. These individuals will be selected based on their expertise in either the clinical management of HIV infection and/or their expertise with clinical trials. The DMC will be chaired by a senior investigator with experience in the regulatory aspects of clinical trials. The DMC will meet at **12, 24, 48, and 60** weeks after the enrollment of the first subject, and at 60 weeks after the enrollment of the last subjects. The DMC will review study progress, efficacy data, all interim and total adverse events, and unanticipated problems involving risk to participants. Reviews will be communicated to the CHR, study sponsor, and/or federal agencies (as appropriate).

21.2 This study requires a Data and Safety Monitoring Board:

- ● Yes
- ○ No or not sure

If **yes**, press **SAVE and CONTINUE** to move to the next section of the application.

21.3 If No, provide rationale:

- ○ Social/Behavioral research
- ○ Phase I trial
- ○ Treatment IND/Compassionate Use Trial
- ○ Other (explain below)

If **Other,** explain:

22.0 Data and Safety Monitoring Board

22.1 Provide details from the Data and Safety Monitoring Board's charter, including meeting frequency, and affiliations and qualifications of members:

We have developed an independent Data Monitoring Committee (DMC). The DMC is composed of 3 independent individuals from the scientific community selected by the principal investigator and co-principal investigator. These individuals will be selected based on their expertise in either the clinical management of HIV infection and/or their expertise with clinical trials. The DMC will be chaired by a senior investigator with experience in the regulatory aspects of clinical trials. The DMC will meet at **12, 24, 48, and 60** weeks after the enrollment of the first subject, and at 60 weeks after the enrollment of the last subjects. The DMC will review study progress, efficacy data, all interim and total adverse events, and unanticipated problems involving risk to participants. Reviews will be communicated to the CHR, study sponsor, and/or federal agencies (as appropriate).

22.2 All of the members of the Data and Safety Monitoring Board are independent of the sponsor:

● Yes ○ No

23.0 Confidentiality and Privacy

23.1 Plans for maintaining privacy in the research setting:

Participation in research will involve a loss of privacy. Research records will be handled as confidentially as possible, but complete confidentiality cannot be guaranteed. All research records will be coded with an ID number. No names will be used. No individual identities will be used in any reports or publications resulting from this study.

23.2 Possible consequences to subjects resulting from a loss of privacy:

If a subject's HIV status is to be revealed publicly this could risk reputation, social stigma, insurability and even partner violence. These considerations are factored into the very strict measures used to protect the confidentiality of study participants.

23.3	Study data are:

- ◻ Derived from the Integrated Data Repository (IDR) or The Health Record Data Service (THREDS) at SFGH
- ■ Derived from a medical record (identify source below)
- ■ Added to the hospital or clinical medical record
- ◻ Created or collected as part of health care
- ■ Used to make health care decisions
- ■ Obtained from the subject, including interviews, questionnaires
- ◻ Obtained from a foreign country or countries only
- ◻ Obtained from records open to the public
- ■ Obtained from existing research records
- ◻ None of the above

If **derived from a medical record**, identify source:

HERO

23.4	Identifiers may be included in research records:

● Yes ○ No

If **yes**, check all the identifiers that may be included:

- ■ Names
- ■ Dates
- ■ Postal addresses
- ■ Phone numbers
- ◻ Fax numbers
- ■ Email addresses
- ◻ Social Security Numbers*
- ■ Medical record numbers
- ◻ Health plan numbers
- ◻ Account numbers
- ◻ License or certificate numbers
- ◻ Vehicle ID numbers
- ◻ Device identifiers or serial numbers
- ◻ Web URLs
- ◻ IP address numbers
- ◻ Biometric identifiers
- ◻ Facial photos or other identifiable images
- ◻ Any other unique identifier

* Required for studies conducted at the VAMC

23.5	Identifiable information might be disclosed as part of study activities:

● Yes ○ No

If **yes**, indicate to whom identifiable information may be disclosed:

- ◻ The subject's medical record
- ■ The study sponsor

- ◻ Collaborators
- ■ The US Food & Drug Administration (FDA)
- ■ Others (specify below)
- ◻ A Foreign Country or Countries (specify below)

If **Others**, specify:

UCSF CHR

23.6 Indicate how data are kept secure and protected from improper use and disclosure (check all that apply):

NOTE: Whenever possible, do not store subject identifiers on laptops, PDAs, or other portable devices. If you collect subject identifiers on portable devices, you MUST encrypt the devices.

- ◻ Data are stored securely in My Research
- ◻ Data are coded; data key is destroyed at end of study
- ■ Data are coded; data key is kept separately and securely
- ■ Data are kept in a locked file cabinet
- ■ Data are kept in a locked office or suite
- ■ Electronic data are protected with a password
- ■ Data are stored on a secure network
- ◻ Data are collected/stored using REDCap or REDCap Survey

23.7 Additional measures to assure confidentiality and protect identifiers from improper use and disclosure, if any:

No additional steps will be taken to assure that identities of subjects and any of their health information are kept confidential.

Guidelines issued by HIPAA have been strictly followed to ensure patients' PHI is appropriately secure and confidentiality is maximized. All study material will be locked in secure areas restricted to study personnel. Data in the form of electric files will be password-protected and stored on a secure computer server limited to research activities. Stored study data will be stripped of information that may disclose the study's identity, and the file linking the study subject to the study data will be restricted to a password-protected file on a computer server dedicated to this research project.

23.8 This study may collect information that State or Federal law requires to be reported to other officials or ethically requires action:

○ Yes ● No

Explain:

23.9 This study will be issued a Certificate of Confidentiality:

○ Yes ● No

24.0 Subjects

24.1 Check all types of subjects that may be enrolled:

- ☐ Inpatients
- ■ Outpatients
- ☐ Healthy volunteers
- ☐ Staff of UCSF or affiliated institutions

24.2 Additional vulnerable populations:

- ☐ Children
- ☐ Subjects unable to consent for themselves
- ☐ Subjects unable to consent for themselves (emergency setting)
- ☐ Subjects with diminished capacity to consent
- ☐ Subjects unable to read, speak or understand English
- ☐ Pregnant women
- ☐ Fetuses
- ☐ Neonates
- ☐ Prisoners
- ☐ Economically or educationally disadvantaged persons
- ☐ Investigators' staff
- ☐ Students

Explain why it is appropriate to include the types of subjects checked above in this particular study:

Describe the additional safeguards that have been included in the study to protect the rights and welfare of these subjects and minimize coercion or undue influence:

25.0 Recruitment

25.1 * Methods (check all that apply):

- ■ Study investigators (and/or affiliated nurses or staff) recruit their own patients directly in person or by phone.
- ☐ Study investigators recruit their own patients by letter. Attach the letter for review.
- ☐ Study investigators send a "Dear Doctor" letter to colleagues asking for referrals of eligible patients. If interested, the patient will contact the PI or the PI may directly recruit the patients (with documented permission from the patient). Investigators may give the referring physicians a study information sheet for the patients.
- ☐ Study investigators provide their colleagues with a "Dear Patient" letter describing the study. This letter can be signed by the treating physicians and would inform the patients how to contact the study investigators. The study investigators may not have access to patient names and addresses for mailing
- ■ Advertisements, notices, and/or media used to recruit subjects. Interested subjects initiate contact with study investigators. Attach ads, notices, or media text for review. In section below, please explain where ads will be posted.
- ■ Study investigators identify prospective subjects through chart review. (Study investigators request a Waiver of Authorization for recruitment purposes.)
- ☐ Large-scale epidemiological studies and/or population-based studies: Prospective subjects are identified through a registry or medical records and contacted by someone other than their personal physician. (Study investigators request a Waiver

of Authorization for recruitment purposes.)

- ■ Direct contact of potential subjects who have previously given consent to be contacted for participation in research. Clinic or program develops a CHR-approved recruitment protocol that asks patients if they agree to be contacted for research (a recruitment database) or consent for future contact was documented using the consent form for another CHR-approved study.
- □ Study investigators list the study on the School of Medicine list of UCSF Clinical Trials website or a similarly managed site. Interested subjects initiate contact with investigators.
- □ Study investigators recruit potential subjects who are unknown to them through methods such as snowball sampling, direct approach, use of social networks, and random digit dialing.
- □ Other

If **Other**, explain:

25.2 * How, when, and by whom eligibility will be determined:

Study investigators and coordinator will recruit patients from an ongoing cohort study of over 1000 chronically HIV-infected patients, the "Study of the Consequences of the Protease Inhibitor Era" (SCOPE), of which approximately 100 meet inclusion criteria for this trial. Advantages of using SCOPE to perform this proposed study include: (1) the ability to leverage existing funds to support the proposed research; (2) rapid recruitment of eligible subjects from a well-characterized cohort; (3) reliance on an established infrastructure for patient retention, data collection, laboratory testing, specimen cryopreservation, and data management.

25.3 * How, when, where and by whom potential subjects will be approached:

Recruitment of eligible subjects will be greatly aided by an established prospective, clinic-based cohort study of HIV-infected adults (the "Study of the Consequences of the Protease Inhibitor Era," or SCOPE (S. Deeks, PI). To date, SCOPE has enrolled over 1000 subjects: over 100 are expected to meet criteria outlined in this proposed study. During the enrollment period all SCOPE patients who come in for regular cohort check-ups will be asked if they would like to learn more about the mesalamine trial. Subjects in the SCOPE study have already given consent allowing the SCOPE team to approach them for other studies.

25.4 * Protected health information (PHI) will be accessed prior to obtaining consent:

● Yes ○ No

26.0 Waiver of Consent/Authorization for Recruitment Purposes

(Note: This section partially replaces the old "Request for Waiver of Consent/Authorization for Minimal Risk Research or for Screening for Recruitment" supplement form. Please do not attach the old form to this application.)

This section is now required when study investigators (and/or affiliated nurses or staff) recruit their own patients directly.

26.1 * Study personnel need to access protected health information (PHI) during the

	recruitment process and it is not practicable to obtain informed consent until potential subjects have been identified:

- Yes

If **no**, a waiver of consent/authorization is NOT needed.

26.2	* A waiver for screening of health records to identify potential subjects poses no more than minimal risk to privacy for participants:

- Yes

If **no**, a waiver of authorization can NOT be granted.

26.3	* Screening health records prior to obtaining consent will not adversely affect subjects' rights and welfare:

- Yes

If **no**, a waiver of authorization can NOT be granted.

26.4	* Check all the identifiers that will be collected prior to obtaining informed consent:

- ■ Names
- ■ Dates
- ■ Postal addresses
- ■ Phone numbers
- □ Fax numbers
- ■ Email addresses
- □ Social Security Numbers*
- ■ Medical record numbers
- □ Health plan numbers
- □ Account numbers
- □ License or certificate numbers
- □ Vehicle ID numbers
- □ Device identifiers or serial numbers
- □ Web URLs
- □ IP address numbers
- □ Biometric identifiers
- □ Facial photos or other identifiable images
- □ Any other unique identifier
- □ None

Note: HIPAA requires that you collect the minimum necessary.

26.5	* Describe any health information that will be collected prior to obtaining informed consent:

1. Name

2. Date of Birth

3. Eligibility Screening, such as the following:

- HIV-1 infection, as documented by any licensed ELISA test kit and confirmed by Western blot at any time prior to study entry.
- Stable antiretroviral therapy for at least 6 months.
- Screening CD4+ T cell count below 350 cells/mm^3
- All available CD4+ T cell counts in the last year and at screening <350 cells/mm^3
- Screening plasma HIV RNA levels below level of detection (< 40 copies RNA/mL).
- All available plasma HIV RNA levels within past year below the level of detection. Isolated detectable values < 500 c/ml are allowed if HIV RNA levels before and after this time point are undetectable.
- >90% adherence to therapy within the preceding 30 days, as determined by self-report.
- Both male and female subjects are eligible. Females of childbearing potential must have negative pregnancy test at screening and agree to use a double-barrier method of contraception during the study.

Note: HIPAA requires that you collect the minimum necessary.

26.6 * Describe your plan to destroy the identifiers at the earliest opportunity consistent with the research or provide a health or research justification for retaining the identifiers, or indicate and explain that retention is required by law:

Data collected about subjects whose consent is not obtained will not be retained and will be immediately destroyed. Any electronic files will be securely destroyed and documents containing PHI will be shredded.

27.0 Informed Consent

27.1 * Methods (check all that apply):

■ Signed consent will be obtained from subjects and/or parents (if subjects are minors)
□ Verbal consent will be obtained from subjects using an information sheet or script
□ Electronic consent will be obtained from subjects via the web or email
□ Implied consent will be obtained via mail, the web or email
□ Signed consent will be obtained from surrogates
□ Emergency waiver of consent is being requested for subjects unable to provide consent
□ Informed consent will not be obtained

27.2 * Process for obtaining informed consent:

Prior to any study procedures, subjects will be consented by the principal investigator or research staff. The consent procedure will be performed in a private setting in the clinic. Subjects will be asked to provide authorization for release of personal health information and use of personally unidentified study data for research (HIPAA).

27.3	* How investigators will make sure subjects understand the information provided to them:

Research staff will ensure that candidates understand all the elements of the consent form by addressing questions posed during the consent procedure and by asking for verbal confirmation that the candidate has no additional questions and verbally understands the purpose of the study, study intervention, basic procedures, risks and that participation is voluntary. Subjects will be given as much time as they need to read and understand the consent form. The subject will receive a signed/dated copy of the consent form to keep along with the UCSF Subject Bill of Rights and the HIPAA form to keep.

28.0 Financial Considerations

28.1	Subjects payment or compensation method (check all that apply):

Payments will be (check all that apply):

- □ Subjects will not be paid
- ■ Cash
- □ Check
- □ Gift card
- □ Other:

Specify **Other**:

28.2	Describe the schedule and amounts of payments, including the total subjects can receive for completing the study. If deviating from recommendations in Subject Payment Guidelines, include specific justification below.

Subjects will receive $20 cash per study visit to cover costs related to the study. This is a total of $180 for completion of 9 study visits. There is no payment for the screening visit.

28.3	Costs to Subjects: Will subjects or their insurance be charged for any study procedures?

○ Yes ● No

If **yes,** describe those costs below, and compare subjects' costs to the costs associated with alternative care off-study. Finally, explain why it is appropriate to charge those costs to the subjects.

29.0 CTSI Screening Questions

29.1	* This study will be carried out at one of the UCSF Clinical Research Services (CRS) units or will utilize CRS services:

● Yes ○ No

29.2	This project involves community-based research:

○ Yes ● No
